# The FDA-Approved Drug Cobicistat Synergizes with Remdesivir To Inhibit SARS-CoV-2 Replication *In Vitro* and Decreases Viral Titers and Disease Progression in Syrian Hamsters

**DOI:** 10.1128/mbio.03705-21

**Published:** 2022-03-01

**Authors:** Iart Luca Shytaj, Mohamed Fares, Lara Gallucci, Bojana Lucic, Mahmoud M. Tolba, Liv Zimmermann, Julia M. Adler, Na Xing, Judith Bushe, Achim D. Gruber, Ina Ambiel, Ahmed Taha Ayoub, Mirko Cortese, Christopher J. Neufeldt, Bettina Stolp, Mohamed Hossam Sobhy, Moustafa Fathy, Min Zhao, Vibor Laketa, Ricardo Sobhie Diaz, Richard E. Sutton, Petr Chlanda, Steeve Boulant, Ralf Bartenschlager, Megan L. Stanifer, Oliver T. Fackler, Jakob Trimpert, Andrea Savarino, Marina Lusic

**Affiliations:** a Department of Infectious Diseases, Integrative Virology, Heidelberg University, Heidelberg, Germany; b Federal University of São Paulo, Infectious Diseases Department, São Paulo, Brazil; c Department of Hydrobiology, Veterinary Research Division, National Research Centre, Cairo, Egypt; d Center for Informatics Science (CIS), Nile University, Sheikh Zayed City, Giza, Egypt; e German Center for Infection Research (DZIF), Heidelberg, Germany; f Pharmaceutical Division, Ministry of Health and Population, Faiyum, Egypt; g Department of Infectious Diseases, Virology, CIID, Heidelberg University Hospital, Heidelberg, Germany; h Schaller Research Groups, Center of Infectious Diseases, Virology, Heidelberg University Hospital, Heidelberg, Germany; i Institut für Virologie, Freie Universität Berlin, Berlin, Germany; j Institute of Veterinary Pathology, Freie Universität Berlin, Berlin, Germany; k Biomolecular Simulation Center, Department of Pharmaceutical Chemistry, Heliopolis University, Cairo, Egypt; l Department of Infectious Diseases, Molecular Virology, CIID, Heidelberg University, Heidelberg, Germany; m Department of Microbiology and Immunology, Emory University School of Medicine, Atlanta, Georgia, USA; n Department of Biochemistry, Faculty of Pharmacy, Minia University, Minia, Egypt; o Department of Regenerative Medicine, Graduate School of Medicine and Pharmaceutical Sciences, University of Toyama, Toyama, Japan; p Yale School of Medicine, Department of Internal Medicine, Section of Infectious Diseases, New Haven, Connecticut, USA; q Department of Molecular Genetics and Microbiology, College of Medicine, University of Florida, Gainesville, Florida, USA; r Department of Infectious Diseases, Italian Institute of Health, Rome, Italy; Max Planck Institute for Infection Biology

**Keywords:** COVID-19, SARS-CoV-2, spike protein, direct-acting antivirals, cobicistat, remdesivir, drug repurposing

## Abstract

Combinations of direct-acting antivirals are needed to minimize drug resistance mutations and stably suppress replication of RNA viruses. Currently, there are limited therapeutic options against the severe acute respiratory syndrome coronavirus 2 (SARS-CoV-2), and testing of a number of drug regimens has led to conflicting results. Here, we show that cobicistat, which is an FDA-approved drug booster that blocks the activity of the drug-metabolizing proteins cytochrome P450-3As (CYP3As) and P-glycoprotein (P-gp), inhibits SARS-CoV-2 replication. Two independent cell-to-cell membrane fusion assays showed that the antiviral effect of cobicistat is exerted through inhibition of spike protein-mediated membrane fusion. In line with this, incubation with low-micromolar concentrations of cobicistat decreased viral replication in three different cell lines including cells of lung and gut origin. When cobicistat was used in combination with remdesivir, a synergistic effect on the inhibition of viral replication was observed in cell lines and in a primary human colon organoid. This was consistent with the effects of cobicistat on two of its known targets, CYP3A4 and P-gp, the silencing of which boosted the *in vitro* antiviral activity of remdesivir in a cobicistat-like manner. When administered *in vivo* to Syrian hamsters at a high dose, cobicistat decreased viral load and mitigated clinical progression. These data highlight cobicistat as a therapeutic candidate for treating SARS-CoV-2 infection and as a potential building block of combination therapies for COVID-19.

## INTRODUCTION

The ongoing pandemic of severe acute respiratory syndrome coronavirus 2 (SARS-CoV-2) poses the challenge of quick development of antiviral therapies. SARS-CoV-2 is an enveloped, positive-sense, RNA virus of the *Coronaviridae* family, which includes other human-infecting pathogens such as SARS-CoV and Middle East respiratory syndrome coronavirus (MERS-CoV) (1). Currently, there are no widely approved antivirals to treat infection with coronaviruses. Large-scale clinical trials have identified immune-modulating agents (e.g., dexamethasone [2]) as potential treatments for coronavirus disease 2019 (COVID-19). However, few direct-acting antiviral agents have shown clinical benefit so far (3). On the other hand, a set of antiviral drugs initially shown to inhibit SARS-CoV-2 replication (remdesivir, chloroquine/hydroxychloroquine) has been unable to reproducibly decrease mortality in randomized, placebo-controlled trials (4, 5).

Complete inhibition of SARS-CoV-2 replication will likely require combinations of antivirals, in line with previous evidence on other RNA viruses (6). Candidate inhibitors have been proposed to target several critical steps of SARS-CoV-2 replication, including viral entry, polyprotein cleavage by viral proteases, transcription, and viral RNA replication (7). SARS-CoV-2 entry is mediated by the spike glycoprotein (S-glycoprotein), which binds through its S1 subunit to the cellular receptor angiotensin-converting enzyme 2 (ACE2). Upon binding, viral entry requires a proteolytic activation of the S2 subunit leading to the fusion of the viral envelope with the host cell membrane (8). The study of candidate inhibitors of SARS-CoV-2 entry has mainly focused on monoclonal antibodies and small molecules to target the association of the receptor binding domain (RBD) of the S-glycoprotein to ACE2 (9). The intensively studied antimalarials chloroquine and hydroxychloroquine have been suggested to impair SARS-CoV-2 entry *in vitro* both by decreasing the binding of the RBD to ACE2 and by decreasing endosomal acidification (10). However, their antiviral activity has not been confirmed in randomized clinical studies (11, 12).

Upon viral membrane fusion, the viral RNA is released to the cytosol and translated into two large polyproteins that are cleaved into nonstructural proteins (nsp’s) by two viral proteases, the main protease (3CL_pro_) and the papain-like protease (PL_pro_). A large body of work to identify antivirals against SARS-CoV-2 has focused on research on these viral proteases (13). Recently, the oral 3CL_pro_ inhibitor PF-07321332 proved able to decrease viral replication and disease progression in a mouse model of the infection (14).

The nsp’s generated by polyprotein cleavage by the viral proteases support the transcription and replication of the viral genome, which are catalyzed by the activity of the RNA-dependent RNA polymerase (RdRp). Owing to its crucial role and high evolutionary conservation, this viral enzyme represents a very attractive therapeutic target, which has so far been exploited by repurposing the anti-Ebola virus drug remdesivir (15) and the anti-influenza drug molnupiravir (16, 17).

A major limitation hampering the development of combined antiviral strategies against SARS-CoV-2 is the paucity of data available on drug interactions. Initial guidelines and *in vitro* results have discouraged the combined use of potentially effective compounds, such as remdesivir and chloroquine/hydroxychloroquine, on the basis of possible antagonism (18) or interference of chloroquine/hydroxychloroquine with remdesivir metabolism, through the efflux pump P-glycoprotein (P-gp) (19, 20). On the other hand, extensive first-pass metabolism by the liver hampers the oral bioavailability of remdesivir and circumscribes its use to intravenous administration, thus limiting both its scalability and, likely, antiviral efficacy (21). Interestingly, a report indicates that the stability of remdesivir in microsomes can be significantly enhanced by the cytochrome P4503A (CYP3A) inhibitor cobicistat (22). More broadly, CYPs and P-gp are responsible for the breakdown and clearance of a large majority of drugs. For this reason, compounds inhibiting CYP function find extensive use in combination therapies (23–25), including the use of ritonavir as a booster for the 3CL_pro_ inhibitor PF-07321332 (14).

Here, we demonstrate that the FDA-approved CYP3A inhibitor cobicistat, typically used as a booster of HIV-1 protease inhibitors (24), can block SARS-CoV-2 replication *in vitro* in cell lines of lung and gut origin and *in vivo* in Syrian hamsters. While cobicistat was identified through *in silico* screening by several groups as a potential inhibitor of 3CL_pro_, our data show that cobicistat can inhibit the fusion of the viral S-glycoprotein to the cell membrane. The antiviral concentrations of cobicistat are above those typically used for HIV-1 treatment but well tolerated *in vitro* and *in vivo*. In combination with remdesivir, cobicistat exhibits a synergistic effect in rescuing cell viability and abrogating viral replication both in cell lines and in a primary colon organoid. Moreover, we show that the combination of cobicistat and remdesivir also exerts antiviral effects *in vivo.* Overall, our data show that cobicistat has a dual activity both as an antiviral drug and as a pharmacoenhancer, thus potentially constituting a basis for combined therapies aimed at complete suppression of SARS-CoV-2 replication.

## RESULTS

### *In silico* and *in vitro* analyses identify cobicistat as a candidate inhibitor of SARS-CoV-2 replication.

**FIG 1:**
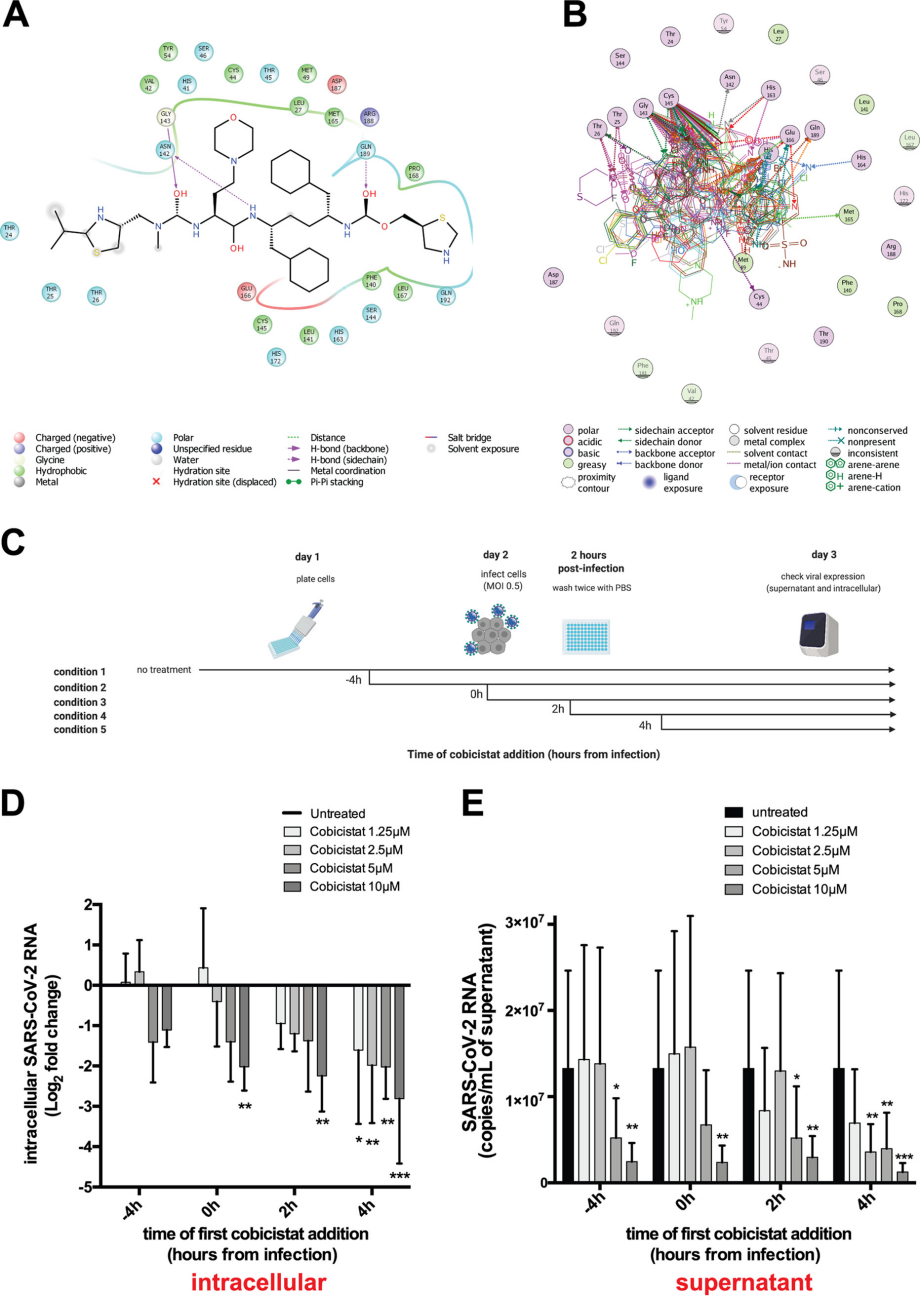
Cobicistat is a candidate inhibitor of SARS-CoV-2 replication. (A and B) *In silico* docking analysis of the putative mode and energy of binding of cobicistat to SARS-CoV-2 3CL_pro_. (A) Docking pose showing the ligand interaction of cobicistat to the active site of 3CL_pro_ and the formation of hydrogen bonds to Asn142, Gly143, and Gln189 of 3CL_pro_. (B) Overlay of crystal structures of SARS-Cov-2 3CL_pro_ showing the amino acids important for the binding of cobicistat to the active site of the enzyme. Residues of the catalytic dyad (Cys145 and His41) of 3CL_pro_ were among the highest contributors to noncovalent binding to cobicistat. The source and list of structures used are detailed in Materials and Methods. (C) Schematic representation of time course experiments evaluating *in vitro* inhibition of SARS-CoV-2 replication by cobicistat (created with BioRender). (D and E) Effect of various concentrations of cobicistat, added according to the scheme of panel C, on intracellular and supernatant SARS-CoV-2 RNA content in Calu-3 cells. Viral RNA content was measured by qPCR using the 2019-nCoV_N1 primer set (Centers for Disease Control and Prevention). Fold change values in intracellular RNA (D) were calculated by the delta-delta *C_T_* method (74), using the Tata-binding protein (TBP) gene as housekeeper control. Expression levels in supernatant (E) were quantified using an *in vitro*-transcribed standard curve generated as described in Materials and Methods. Data are expressed as mean with standard deviation (SD) and were analyzed by two-way ANOVA followed by Dunnett’s posttest (*n* = 3 independent experiments). *, *P* < 0.05; **, *P* < 0.01; ***, *P* < 0.001.

To identify potential inhibitors of SARS-CoV-2 replication, we performed a structure-based virtual screening of the DrugBank library of compounds approved for clinical use. Candidate drugs were ranked based on their docking score to the substrate-binding site of 3CL_pro_, i.e., the site essential for the proteolytic function. Our results highlighted 17 top candidate inhibitors, including compounds used to treat parasitic as well as viral infections (Table 1). Among the latter, the HIV-1 protease inhibitor nelfinavir, which was one of the top-scoring compounds in our analysis, was previously shown to decrease SARS-CoV replication *in vitro* (26) (Table 1). Two additional drugs used for treatment of HIV-1 displayed top docking scores, i.e., the protease inhibitor tipranavir and the CYP3A inhibitor cobicistat. The latter was a particularly interesting candidate, given its activity as a booster for HIV-1 protease inhibitors (24), which renders it a promising candidate for combination therapies. Additional *in silico* investigation of the binding poses of cobicistat to the 3CL_pro_ of SARS-CoV-2 corroborated the potential affinity of this drug for the viral protease (Fig. 1A and B). Moreover, our results were in line with similar independent analyses of other groups that had identified cobicistat as a potential SARS-CoV-2 inhibitor through screenings *in silico* (27–29) and using a reporter model of SARS-CoV-2 replication (30).

**TABLE 1:** Top-scoring list of FDA-approved drugs predicted to bind 3CL_pro_ *in silico*^*a*^

DrugBank ID	Drug group(s)	Generic name	Main indication	Docking score
DB01362	Approved	Iohexol	Contrast agent	−11.72
DB09134	Approved	Ioversol	Contrast agent	−11.03
DB12407	Approved; investigational	Iobitridol	Contrast agent	−10.22
DB12615	Approved; investigational	Plazomicin	Antibiotic for urinary tract infections	−9.43
DB00932	Approved; investigational	Tipranavir	HIV protease inhibitor	−8.06
DB00220	Approved	Nelfinavir	HIV protease inhibitor	−7.91
DB08909	Approved	Glycerol phenylbutyrate	Nitrogen-binding agent for management of urea cycle disorders	−7.86
DB00905	Approved; investigational	Bimatoprost	Analog of prostaglandin F2α for treatment of glaucoma	−7.67
DB08889	Approved; investigational	Carfilzomib	Proteasome inhibitor (anticancer)	−7.54
**DB09065**	**Approved**	**Cobicistat**	**CYP3A inhibitor for boosting HIV-1 protease inhibitors**	−**7.12**
DB04868	Approved; investigational	Nilotinib	Tyrosine kinase inhibitor for treatment of chronic myelogenous leukemia	−7.05
DB01288	Approved; investigational	Fenoterol	Beta adrenergic agonist for asthma treatment	−7.05
DB00482	Approved; investigational	Celecoxib	Nonsteroidal anti-inflammatory drug	−6.80
DB13931	Approved	Netarsudil	Rho kinase inhibitor for treatment of glaucoma	−6.75
DB11611	Approved	Lifitegrast	Anti-inflammatory for treatment of keratoconjunctivitis sicca	−6.45
DB11979	Approved; investigational	Elagolix	Gonadotropin-releasing hormone antagonist for treatment of endometriosis pain	−5.72
DB01116	Approved; investigational	Trimethaphan	Nicotinic antagonist used to counteract hypertension	−5.70

a The DrugBank library of compounds was screened by molecular docking based on the predicted binding mode and affinity of each compound to the allosteric active site of SARS-CoV-2 3CL_pro_. Docking scores were calculated using Glide (66). The data regarding cobicistat are shown in bold.

We then tested the effect of cobicistat on SARS-CoV-2 replication *in vitro*. To this purpose, we conducted a time course analysis of the effect of different concentrations of cobicistat on intracellular viral RNA replication and virus release in the culture supernatant of Calu-3 cells (Fig. 1C to E; also see Fig. S1A and B in the supplemental material). Analysis of virus RNA amounts by quantitative PCR (qPCR) showed a dose-dependent inhibitory effect of low-micromolar concentrations of cobicistat (Fig. 1D and E; Fig. S1A and B). This effect was visible in both supernatants and cellular extracts and was reproducible when samples were assayed with two different sets of primers (i.e., N1 and N2 primer sets [Table 2] as recommended by the Centers for Disease Control and Prevention [Fig. 1D and E; Fig. S1A and B]). In line with these results, cobicistat was also able to decrease the levels of replication-competent virus in the supernatant of Vero E6 cells (Fig. S1C).

**TABLE 2:** List of qPCR primers used in the study

Name^*a*^	Sequence	Source
2019-nCoV_N1-Forward	GAC CCC AAA ATC AGC GAA AT	https://www.cdc.gov/coronavirus/2019-ncov/lab/rt-pcr-panel-primer-probes.html
2019-nCoV_N1-Reverse	TCT GGT TAC TGC CAG TTG AAT CTG	
2019-nCoV_N2-Forward	TTA CAA ACA TTG GCC GCA AA	https://www.cdc.gov/coronavirus/2019-ncov/lab/rt-pcr-panel-primer-probes.html
2019-nCoV_N2-Reverse	GCG CGA CAT TCC GAA GAA	
Hum Cyp3A4-Forward	TGA TGG CTC TCA TCC CAG AC	
Cyp3A4-Reverse	AGC CCC ACA CTT TTC CAT AC	
AGM Cyp3A4-Forward	TGA TGG ACC TCA TCC CAG AC	
Hum Cyp3A5-Forward	CGA CAA ACA AAA GCA CCG AC	
Hum Cyp3A5-Reverse	TTA TTG ACT GGG CTG CGA G	
AGM Cyp3A5-Forward	CGA CAA ACA AAA GCA CCG AG	
AGM Cyp3A5-Reverse	TAA TTG ATT GGG CCA CGA G	
P-gp (MDR1)-F	CCC ATC ATT GCA ATA GCA GG	Gao et al., 2015 (83)
P-gp (MDR1)-R	TGT TCA AAC TTC TGC TCC TGA	
TBP-F	CCA CTC ACA GAC TCT CAC AAC	Stanifer et al., 2020 (47)
TBP-R	CTG CGG TAC AAT CCC AGA ACT	

a Abbreviations: Hum, human; AGM, African green monkey (Vero E6 cells).

10.1128/mbio.03705-21.1 FIG S1 Validation of the antiviral potential of cobicistat. (A and B) Validation of the antiviral effects of cobicistat in Calu-3 cells shown in Fig. 1D and E using a distinct set of primers (2019-nCoV_N2 primer set; Centers for Disease Control and Prevention). Fold change values in intracellular RNA (A) were calculated by the delta-delta *C_T_* method (74), using the Tata-binding protein (TBP) gene as a housekeeper control. Expression levels in supernatant (B) were quantified using an *in vitro*-transcribed standard curve generated as described in Materials and Methods. (C) Effect of cobicistat on the levels of replication-competent virus in Vero E6 cells. Vero E6 cells were plated at 125,000 cells per well and infected with SARS-CoV-2 at an 0.05 MOI. Two hours postinfection, cells were left untreated or treated with various concentrations of cobicistat. Supernatants were collected at 24 h postinfection and used to overlay cells in the plaque assay. Data are expressed as mean with SD (*n* = 3) and were analyzed by two-way ANOVA followed by Dunnett’s posttest for panels A and B or one-way ANOVA followed by Tukey’s posttest for panel C. *, *P* < 0.05; **, *P* < 0.01. Download FIG S1, PDF file, 0.4 MB. Copyright © 2022 Shytaj et al. 2022 Shytaj et al. https://creativecommons.org/licenses/by/4.0/ This content is distributed under the terms of the Creative Commons Attribution 4.0 International license.

Taken together, these data show that cobicistat has a direct antiviral effect on SARS-CoV-2 replication *in vitro*.

### The antiviral concentration range of cobicistat is well tolerated *in vitro* and compatible with plasma levels achievable in humans and mice.

**FIG 2:**
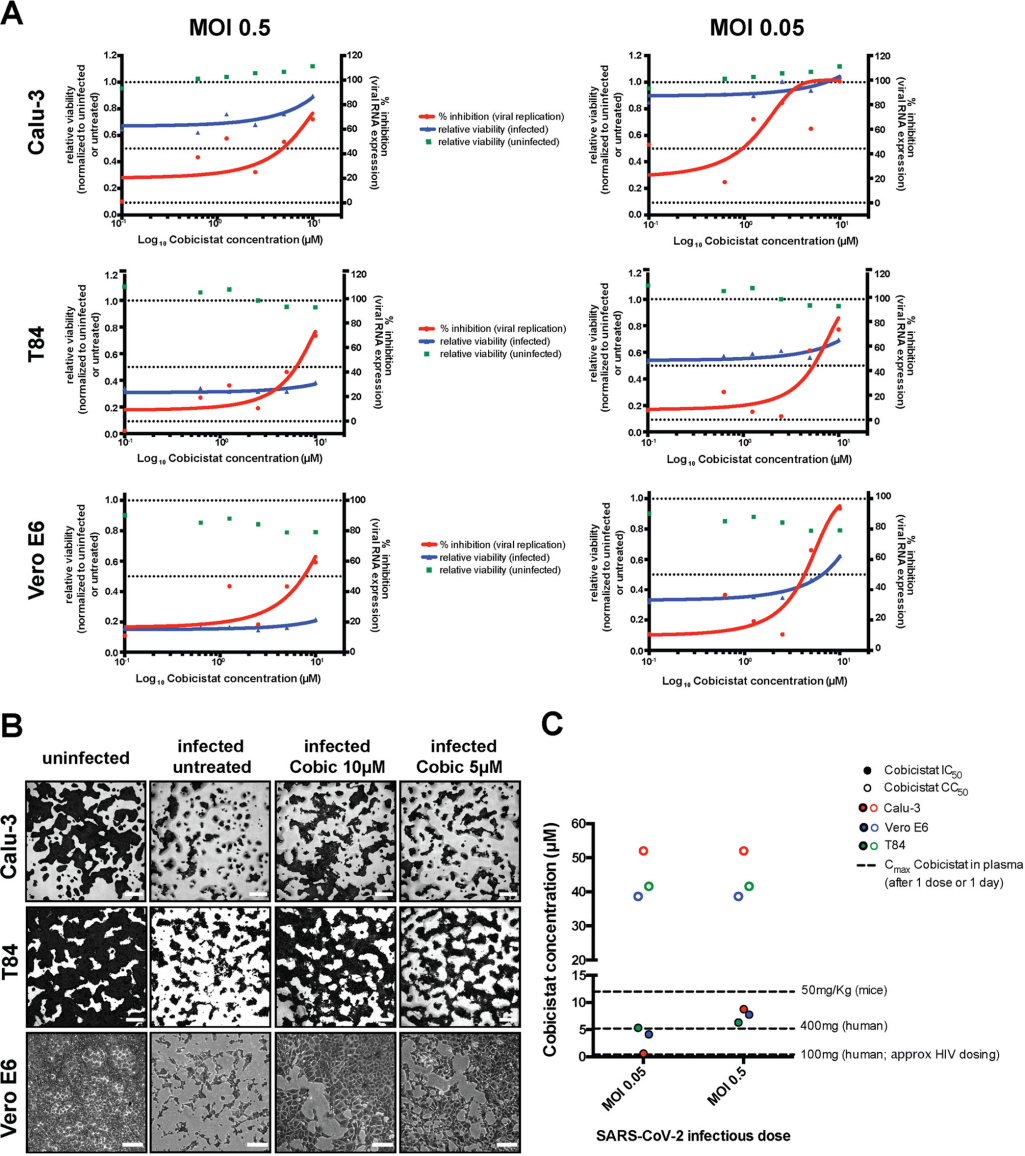
Cobicistat decreases replication of SARS-CoV-2 and rescues viability of infected cells in multiple *in vitro* models. (A and B) Effect of serial dilutions of cobicistat on SARS-CoV-2 RNA concentration in supernatants (A) and on the viability of infected and uninfected cell lines of lung (Calu-3), gut (T84), and kidney (Vero E6) origin (A and B). Cells were infected with SARS-CoV-2 at two different MOIs (0.05 and 0.5) and left untreated or treated with cobicistat 2 h postinfection. Forty-eight hours postinfection, supernatants were collected and viral RNA was assayed by qPCR while cellular viability was measured by MTT assay (A) or by crystal violet staining (B). Inhibition of viral replication was calculated as described in Materials and Methods while viability data were normalized to the uninfected or to the untreated control. Half-maximal inhibitory concentration (IC_50_) values were calculated by nonlinear regression. Each point in panel A represents a mean from 3 independent experiments. Pictures in panel B are derived from infections at MOI 0.5 (Calu-3 and T84 cells) or MOI 0.05 (Vero E6 cells). (C) Comparison between the IC_50_ and CC_50_ values of cobicistat determined *in vitro* and the peak plasma levels detectable in mice (Pharmacology Review of Cobicistat - application number: 203-094) and in humans (33, 81) after administration of a single dose of the drug. Determination of *in vitro* CC_50_ values is based on the data shown in Fig. S2.

We next analyzed more thoroughly the antiviral effects of cobicistat using three cell lines of different origin, i.e., Calu-3 cells (human lung), Vero E6 cells (African green monkey kidney), and T84 cells (human gut), to reflect various known or putative tissue compartments of SARS-CoV-2 replication. Each cell line was infected using two different multiplicities of infection (MOI; 0.05 and 0.5) and left untreated or treated with various concentrations of cobicistat 2 h postinfection. In all cell lines, cobicistat showed a dose-dependent effect in decreasing viral RNA release in supernatant (Fig. 2A). In line with this, the higher concentrations of cobicistat tested (5 to 10 μM) were able to partially rescue viability of infected cells, as shown by both the MTT [3-(4,5-dimethylthiazol-2-yl)-2,5-diphenyl tetrazolium bromide] and crystal violet assay (Fig. 2A and B), while being well tolerated by uninfected cells (Fig. 2A; Fig. S2). Overall, the range of 50% inhibitory concentrations (IC_50_) of cobicistat (0.58 to 8.76 μM) was dependent on the MOI of the infection and on the cell type but always far below the half-cytotoxic concentration (CC_50_) range of the drug on the same cell lines (38.66 to 53 μM). We then compared our *in vitro* results with previously known pharmacokinetic properties of cobicistat in humans and mice. Interestingly, maximum plasma concentrations achievable through standard dosing of cobicistat (150 mg/day as a booster for HIV-1 protease inhibitors) (24) were well below (≈1 μM) most IC_50_ values obtained in our experiments (Fig. 2C). This result is in line with the lack of effect, or only partial benefit, reported when cobicistat-boosted darunavir was tested as a treatment for SARS-CoV-2 patients (31, 32). On the other hand, plasma levels achievable with a higher dosage of cobicistat, which was well tolerated in clinical trials (400 mg/day) (33), were above IC_50_ values calculated when cells were infected using a 0.05 MOI (Fig. 2C). Moreover, plasma levels achievable in mice with a cobicistat dosage shown to be safe in this animal model (50 mg/kg of body weight) were clearly above all IC_50_ values calculated in our experiments, while remaining below the CC_50_s (Fig. 2C).

10.1128/mbio.03705-21.2 FIG S2 Effect of various concentrations of cobicistat on the viability of the cell lines employed in the study. (A to C) Uninfected cell lines of lung (Calu-3) (A), gut (T84) (B), and kidney (Vero E6) (C) origin were left untreated or treated with serial dilutions of cobicistat. Forty-eight hours posttreatment, cellular viability was measured by MTT assay. Data, expressed as mean ± SD from three independent experiments, were normalized to the untreated control, and CC_50_ values were calculated by nonlinear regression. Download FIG S2, PDF file, 0.4 MB. Copyright © 2022 Shytaj et al. 2022 Shytaj et al. https://creativecommons.org/licenses/by/4.0/ This content is distributed under the terms of the Creative Commons Attribution 4.0 International license.

Overall, our data show that nontoxic concentrations of cobicistat can consistently decrease SARS-CoV-2 replication in various cellular infection models. Moreover, these data suggest that higher doses of cobicistat, compared to the standard of care for HIV-1/AIDS, appear to be required to achieve plasma levels within the concentration range predicted to display antiviral activity.

### Cobicistat decreases S-glycoprotein-mediated syncytium formation and fusion *in vitro*.

To characterize the mechanism of the antiviral effects of cobicistat, we analyzed the catalytic activity of 3CL_pro_ using a previously described fluorescence resonance energy transfer (FRET) assay (34). While treatment with known inhibitors of 3CL_pro_, such as GC376 and MG-132, potently reduced the catalytic activity of the enzyme, cobicistat was surprisingly inactive (Fig. S3A). Among the top-scoring compounds in our docking analysis (Table 1), only tipranavir proved able to partially inhibit 3CL_pro_ activity, although at relatively high concentrations (half-maximal effective concentration [EC_50_] 47 μM; Fig. S3A and Table 3). A lack of binding stability of the ligands within the active site of 3CL_pro_ might explain the discrepancy between the FRET experimental results and the previous *in silico* predictions indicating cobicistat as a 3CL_pro_ inhibitor. In particular, when assessment of conformational entropy was included in the molecular dynamics analysis (35), the binding of cobicistat tended to be less stable than that of other ligands (Table 3; Movie S1), and the predicted binding energies to 3CL_pro_ of the examined ligands reflected more closely the EC_50_ values calculated by FRET (Fig. S3B; Table 3).

**TABLE 3:** *In silico* and *in vitro* affinity of cobicistat and other putative inhibitors to SARS-CoV-2 3CL_pro_^*a*^

Ligand	Δ*G* solvation	Δ*E* interactions	−TΔS	Δ*G* total	FRET-determined EC_50_ (3CL_pro_)
GC376	35.9	−69.3	18.2	−15.2	0.11 μM
X77 (docked)	51.1	−88.9	25.6	−12.2	NA
X77 (native)	34.4	−62.8	17	−11.4	NA
Tipranavir	28.8	−54.4	19.5	−6	47 μM
Lopinavir	28.3	−61.9	28.6	−5	219 μM
MG-132	19.1	−41.6	18	−4.5	18 μM
Darunavir	10.9	−17.8	17.1	10.3	Could not be calculated
Cobicistat	82.9	−112.8	44	14.2	Could not be calculated
Nelfinavir	112.7	−152.4	81.3	41.6	Could not be calculated

a *In silico* binding stability of the ligands to 3CL_pro_ was estimated by molecular dynamics including the contribution of entropy, as previously described (71). Binding free energies (ΔGb) were calculated as described in Materials and Methods. *In vitro* inhibition of 3CL_pro_ was measured by FRET assay, as shown in Fig. 3A. Data were normalized to the untreated control, and half-maximal effective concentration (EC_50_) values for each ligand were calculated by nonlinear regression. NA, not available.

10.1128/mbio.03705-21.3 FIG S3 Binding prediction and testing of potential inhibitors of 3CL_pro_. (A) *In vitro* screening of putative inhibitors of the enzymatic activity of 3CL_pro_. The activity of 3CL_pro_ was measured by FRET assay (34) and normalized over the untreated condition. Apart from cobicistat, compounds tested included HIV-1 protease inhibitors highlighted by our molecular docking (nelfinavir and tipranavir [Table 1]) or previously administered in clinical trials as SARS-CoV-2 therapeutics (darunavir [31] and lopinavir [93]), as well as two positive controls known to inhibit 3CL_pro_ activity (MG-132 and GC376 [94]). (B) *In silico* prediction of the binding stability of the ligands to 3CL_pro_ as estimated by molecular dynamics. The previously described inhibitors of 3CL_pro_ X77 (65) and GC376 and MG-132 (94) were included as comparisons. Download FIG S3, PDF file, 0.5 MB. Copyright © 2022 Shytaj et al. 2022 Shytaj et al. https://creativecommons.org/licenses/by/4.0/ This content is distributed under the terms of the Creative Commons Attribution 4.0 International license.

10.1128/mbio.03705-21.10 MOVIE S1 Animation of the molecular dynamics trajectory of cobicistat bound to the 3CL_pro_ of SARS-CoV-2. Molecular dynamics analysis performed including the contribution of entropy, as previously described (71), shows instability of the binding of cobicistat, characterized by its constant change in orientation. Download Movie S1, MPG file, 16.4 MB. Copyright © 2022 Shytaj et al. 2022 Shytaj et al. https://creativecommons.org/licenses/by/4.0/ This content is distributed under the terms of the Creative Commons Attribution 4.0 International license.

We thus proceeded to analyze the possible impact of cobicistat on other key viral proteins. To reduce the bias of the analysis, while retaining a representative model of the infection, we performed Western blot analysis of Vero E6 cell lysates using previously validated patient sera to detect viral proteins (36). The results showed the reduction of a high-molecular-weight band (≈250 kDa) when infected cells were incubated with low-micromolar concentrations of cobicistat (Fig. S4A). Based on the known molecular weights of SARS-CoV-2 proteins, we postulated that the patterns detected with patient sera corresponded to dimers/trimers of the S-glycoprotein (37, 38) and to the nucleoprotein (N-protein) (38) of the virus. To confirm this hypothesis, we performed Western blot analysis using monoclonal antibodies directed against the S- and N-protein (Fig. 3A). The results suggested (Fig. 3A) that cobicistat might impact the cleaved form (≈100 kDa) of the S-glycoprotein (37, 39), which is responsible for SARS-CoV-2 fusion to the host cell and subsequent viral entry (8) (Fig. 3B). To isolate the possible effect of cobicistat on S-glycoprotein-mediated fusion, we first used a cellular assay measuring syncytium formation in Vero E6 cells transfected with the S-glycoprotein. The results showed decreased syncytium formation when cells were incubated with cobicistat or when sera from SARS-CoV-2 patients were used as controls to block S-glycoprotein fusion (Fig. 3C and D; Fig. S4B). In line with the results obtained in infected cells, Western blot and immunofluorescence (IF) assays on transfected Vero E6 cells showed significantly decreased levels of the cleaved S-glycoprotein upon cobicistat treatment (Fig. S4C and D). In addition, both analyses showed that the effect of cobicistat on the total levels of the S-glycoprotein, as well as on its relative cellular distribution, was not statistically significant (Fig. S4D to F), further indicating a preferential effect of the drug on the S-glycoprotein membrane fusion ability.

**FIG 3:**
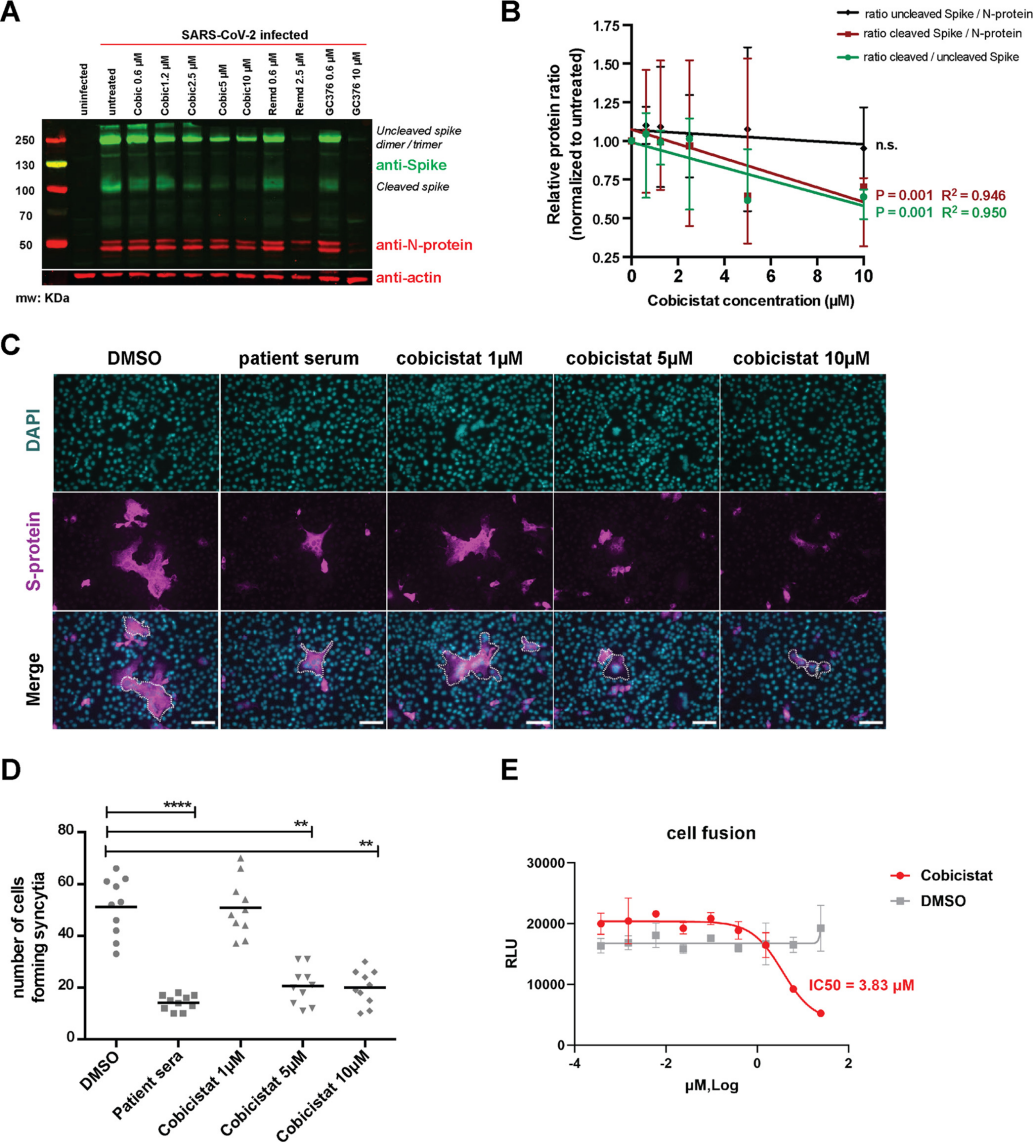
Cobicistat decreases SARS-CoV-2 S-protein content and fusion to target cells. (A and B) Effect of cobicistat on the expression of S- and N-proteins in SARS-CoV-2-infected Vero E6 cells. Cells were infected at 0.5 MOI and left untreated or treated, 2 h postinfection, with various concentrations of cobicistat, of the RdRp inhibitor remdesivir, or of the 3CL_pro_ inhibitor GC376. Cells were harvested 24 h posttreatment and subjected to protein extraction and subsequent analysis by Western blotting. Expression of S- and N-proteins, and expression of the housekeeping protein actin-β, was detected using primary monoclonal antibodies followed by incubation with fluorescence-conjugated secondary antibodies and detection on a Li-Cor Odyssey CLx instrument (B). Relative protein levels were quantified using Fiji‐Image J (78) and normalized to the untreated control. Data (mean ± range of three independent experiments) were analyzed by linear regression. n.s., not significant. (C and D) Effect of cobicistat on S-protein-mediated syncytium formation. Vero E6 cells were transfected with the SARS-CoV-2 S-protein and left untreated or treated with various concentrations of cobicistat or with sera isolated from convalescent SARS-CoV-2 patients (1:100 dilution). Syncytium formation was examined 24 h posttransfection by immunofluorescence (IF) staining for DAPI and S-protein (C) and quantified as the number of cells forming syncytia (D). (E) Effect of cobicistat treatment on S-glycoprotein-mediated fusion. TZM-bl cells stably expressing the S-glycoprotein were incubated with different concentrations of cobicistat for 1 h and mixed with cells stably expressing human ACE2 (40). Cell fusion was assessed by measuring firefly luciferase activity after 24 h. RLU, relative light units. Data in panels C and D were analyzed using the nonparametric Kruskal-Wallis test followed by Dunn’s posttest. Horizontal lines represent mean values. **, *P* < 0.01; ****, *P* < 0.0001. Scale bar = 50 μm.

10.1128/mbio.03705-21.4 FIG S4 Validation of the impact of cobicistat treatment on S-protein expression and fusion. (A) Effect of cobicistat treatment on SARS-CoV-2 protein expression in infected Vero E6 cells. Cells were infected at an 0.5 MOI and left untreated or treated, 2 hours postinfection, with various concentrations of cobicistat, of the RdRp inhibitor remdesivir, or of the 3CL_pro_ inhibitor GC376. Cells were harvested 24 h posttreatment and subjected to protein extraction and Western blot analysis. Expression of viral proteins was detected using sera from convalescent SARS-CoV-2 patients (at 1:200 dilution) followed by incubation with a fluorescence-conjugated anti-human secondary antibody. Expression of the housekeeping protein β-actin was detected using a specific primary monoclonal antibody. Fluorescent signals were measured using a Li-Cor Odyssey CLx instrument. (B to D) Quantification of the effect of cobicistat on syncytium formation and S-glycoprotein isoform content. Vero E6 cells were transfected with the SARS-CoV-2 S-protein and left untreated or treated with various concentrations of cobicistat or with sera isolated from convalescent SARS-CoV-2 patients (1:100 or 1:500 dilution). Syncytium formation (B) was examined 24 h posttransfection by immunofluorescence (IF) staining for DAPI and S-protein (as shown in Fig. 3D) and quantified as the number of cells forming syncytia. Expression of total or cleaved S-glycoprotein was assessed by Western blotting (C and D) or IF (E and F). Relative protein levels in panels C and D were quantified using Image Lab Software (Bio-Rad) and normalized to the untreated control. Images in panel E were selected to display Spike signals in single cells rather than syncytia. Relative S-protein fluorescence in panels E and F was normalized using the DAPI fluorescence. Data were analyzed using the nonparametric Kruskal-Wallis test followed by Dunn’s posttest (B) or by one-way ANOVA followed by Dunnett’s posttest (D and F). Horizontal lines represent mean values. *, *P* < 0.05; **, *P* < 0.01; ***, *P* < 0.001. Scale bar = 50 μm. Download FIG S4, PDF file, 1.5 MB. Copyright © 2022 Shytaj et al. 2022 Shytaj et al. https://creativecommons.org/licenses/by/4.0/ This content is distributed under the terms of the Creative Commons Attribution 4.0 International license.

Finally, to obtain a specific and quantitative estimate of the impact of cobicistat on the S-glycoprotein–ACE2 interaction, we tested the effect of this drug using a previously validated fusion assay (40) based on cell lines stably transfected with the S-glycoprotein and human ACE2. The results showed that cobicistat can inhibit S-glycoprotein fusion with an IC_50_ of 3.8 μM (Fig. 3E). Of note, all assays indicated an effect of cobicistat in the same low-micromolar range of the IC_50_ values calculated on the basis of viral RNA levels in supernatants (Fig. 2A and C).

Overall, these data show that the antiviral effect of cobicistat is not mediated by inhibition of 3CL_pro_ activity but is rather exerted, at least partially, through impairment of S-glycoprotein-mediated fusion.

### Cobicistat potently enhances the antiviral effect of remdesivir in cell lines and a primary colon organoid.

**FIG 4:**
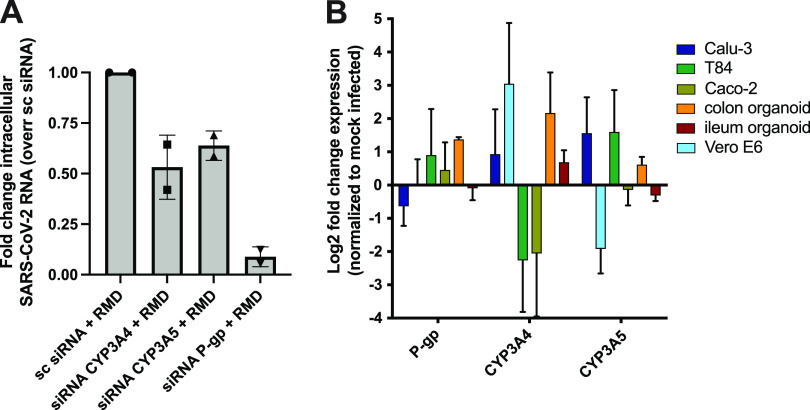
Expression of the metabolic targets of cobicistat and its role in the antiviral activity of remdesivir. (A) Effect of the knockdown of *CYP3A4*, *CYP3A5*, and *P-gp* genes on the antiviral efficacy of remdesivir. Vero E6 cells were transfected with 40 nM siRNAs against either gene target or with nontargeting siRNAs. At 48 h posttransfection cells were infected at MOI 0.05, and 2 h postinfection, they were treated with 0.5 μM remdesivir. Intracellular SARS-CoV-2 RNA expression was analyzed by qPCR 24 h postinfection. (B) Relative expression of *CYP3A4/5* and *P-gp* in SARS-CoV-2-infected or mock-infected cells. Infections were carried out at MOI 0.5 for 48 h, and gene expression was analyzed by qPCR. For both panels, raw data were used to calculate delta *C_T_* values by using the *TBP* gene as housekeeping control. Fold changes were calculated using the delta-delta *C_T_* method, as described in reference 74. Data are expressed as mean ± SD (*n* = 2 for panel A and *n* = 3 for panel B).

We then tested the potential of cobicistat to exert a double activity as a direct inhibitor of SARS-CoV-2 replication and as a pharmacoenhancer of other antivirals. To this aim, we evaluated remdesivir as a candidate compound to synergize with cobicistat. The choice of remdesivir was motivated by its known activity as an inhibitor of SARS-CoV-2 RdRp (41), as well as by its postulated susceptibility to extensive first-pass liver metabolism, potentially mediated by the cellular targets of cobicistat CYP3A and P-gp (21). We thus examined the *in silico*-predicted affinity of remdesivir for the main members of the CYP3A family (CYP3A4 and -5), as well as for P-gp. Multiple machine learning models predicted remdesivir as a potential CYP3A4 substrate (Table 4). Moreover, the SwissADME server (42) predicted remdesivir to be both a CYP3A4 and P-gp substrate with 79% and 88% accuracy, respectively. Similarly, the pkCSM (43) and CYPreact (44) servers also predicted remdesivir to be a substrate, but not an inhibitor, of both P-gp and CYP3A4. Finally, remdesivir displayed high docking scores to the active sites of CYP3A4, CYP3A5, and P-gp, which were comparable to those of ritonavir and cobicistat, i.e., known inhibitors with well-characterized binding (Fig. S5). To confirm these *in silico* predictions, we silenced with small interfering RNAs (siRNAs) the expression of CYP3A4, CYP3A5, or P-gp. Each silencing decreased its specific target mRNA, although the silencing of CYP3A5 also induced a decrease in CYP3A4 levels (Fig. S6). In line with the *in silico* predictions, silencing each of these cobicistat targets enhanced the antiviral activity of remdesivir in Vero E6 cells, with the highest effect observed upon silencing of P-gp (Fig. 4A).

**TABLE 4:** Machine learning prediction of potential binding of remdesivir to CYP3A4^*a*^

Reference	Descriptor feature selection method	Strategy	Classification algorithm	CYP3A4 performance
Korolev et al., 2003 (84)	Principal-component analysis	Binary classification	Kohonen SOM	Accuracy: 76.7%
Yap et al., 2005 (85)	Genetic algorithm	Binary classification	PM-CSVM	MCC: 0.849
Terfloth et al., 2007 (86)	BestFirst or exhaustive search	Binary classification	Multinomial logistic regression, decision tree, SVM	Accuracy: 78.5–82.4%
Michielan et al., 2009 (87)	BestFirst automatic variable selection	Binary classification, multilabel	ct-SVM, ML-KNN, CPG-NN	MCC: 0.44–0.70 (for multilabel classification)
Ramesh and Bharatam, 2012 (88)	Manual	Binary classification	Decision tree	Accuracy: 82%
Nembri et al., 2016 (89)	Genetic algorithm	Binary classification	CART, KNN, N-nearest neighbor	Avg sensitivity, 75%; avg specificity, 78%
Zhang et al., 2012 (90)	Genetic algorithm	Binary classification, multiclass	Decision tree, neural network, ML-KNN, rank SVM	Accuracy: ∼90% on single-label system; ∼80% on multiclass system
Mishra et al., 2010 (91)	Genetic algorithm	Binary classification	Support vector machine	Accuracy: 70.55%
Yamashita et al., 2008 (92)	Manual curation	Binary classification	Decision tree	Accuracy: 84.3%
SwissADME	Manual curation	Binary classification	Support vector machine	Accuracy: 79%
CYPreact	Information gain	Binary	Learning base model	Accuracy: 83%

a The likelihood of remdesivir being a substrate of CYP3A4 was estimated using the algorithms described in references 42 and 44, and their performance was compared to that of previously described algorithms (84–92) as listed in the table. MCC, Matthews correlation coefficient.

10.1128/mbio.03705-21.5 FIG S5 Predicted *in silico* binding of remdesivir to CYP3A4/5 and P-gp. (A to C) Molecular docking analysis of the binding pose and docking score of remdesivir (DB14761) to CYP3A4 (PDB ID: 5VC0) (A), CYP3A5 (PDB ID: 5VEU) (B), and P-gp (PDB ID: 6QEE) (C). Docking scores of the CYP3A and P-gp inhibitors cobicistat (DB09065) and ritonavir (DB00503) are provided as comparisons. All analyses were conducted using the Schrödinger software package. Download FIG S5, PDF file, 1.5 MB. Copyright © 2022 Shytaj et al. 2022 Shytaj et al. https://creativecommons.org/licenses/by/4.0/ This content is distributed under the terms of the Creative Commons Attribution 4.0 International license.

10.1128/mbio.03705-21.6 FIG S6 Expression of the metabolic targets of cobicistat upon silencing with siRNAs. Vero E6 cells were transfected with 40 nM siRNAs targeted against the genes *CYP3A4*, *CYP3A5*, and *P-gp* or with nontargeting siRNAs. At 48 h posttransfection, cells were infected at MOI 0.05, and the expression of each knockdown target was assessed by qPCR 24 h postinfection. Fold changes were calculated against the respective scrambled (sc) siRNA using the delta-delta *C_T_* method, as described in reference 74. Data are expressed as mean ± SD (*n* = 2). Download FIG S6, PDF file, 0.3 MB. Copyright © 2022 Shytaj et al. 2022 Shytaj et al. https://creativecommons.org/licenses/by/4.0/ This content is distributed under the terms of the Creative Commons Attribution 4.0 International license.

To identify the most suitable *in vitro* model for testing the combination of remdesivir and cobicistat, we first examined the relative expression levels of CYP3A4, CYP3A5, and P-gp in different human tissues and cell lines susceptible to SARS-CoV-2 infection (Fig. 4B; Fig. S7). Both transcriptomic and qPCR analysis highlighted liver, gut, and kidney as major compartments of CYP3A4/5 and P-gp expression (Fig. S7A to C), as previously described (25, 45). On the other hand, primary lung tissues were characterized by lower CYP3A4/5 and P-gp expression, while the cell line Calu-3 showed intermediate characteristics, with low CYP3A4 and high P-gp expression (Fig. S7B and C), in line with upregulation of the latter marker in cancer cells (46). Of note, SARS-CoV-2 infection was associated with altered expression of these genes. In this regard, cell lines of gut origin and Vero E6 cells displayed a trend showing opposite expression patterns of CYP3A4 and CYP3A5 upon infection (Fig. 4B). Given their divergent response to the infection, we decided to use both Vero E6 and T84 cells as models for testing cobicistat and remdesivir, to obtain data on the efficacy of the drug combination and on its possible reliance on increased expression of either CYP3A4 or CYP3A5.

10.1128/mbio.03705-21.7 FIG S7 Expression of the metabolic targets of cobicistat in human tissues and cell lines. (A and B) Expression levels of CYP3A4/5 and P-gp in different human tissues from healthy donors (A) or cell lines susceptible to SARS-CoV-2 infection (B). Gene expression data were retrieved from the “Homo sapiens Affymetrix Human Genome U133 Plus 2.0 Array” and from the RNA-Seq “mRNA Gene Level Homo sapiens (ref: Ensembl 75)” data sets. (C) Relative expression of *CYP3A4/5* and *P-gp* genes in uninfected cell lines or human organoids as measured by qPCR. Fold changes were calculated using the delta-delta *C_T_* method, as described in reference 74. Download FIG S7, PDF file, 1.5 MB. Copyright © 2022 Shytaj et al. 2022 Shytaj et al. https://creativecommons.org/licenses/by/4.0/ This content is distributed under the terms of the Creative Commons Attribution 4.0 International license.

**FIG 5:**
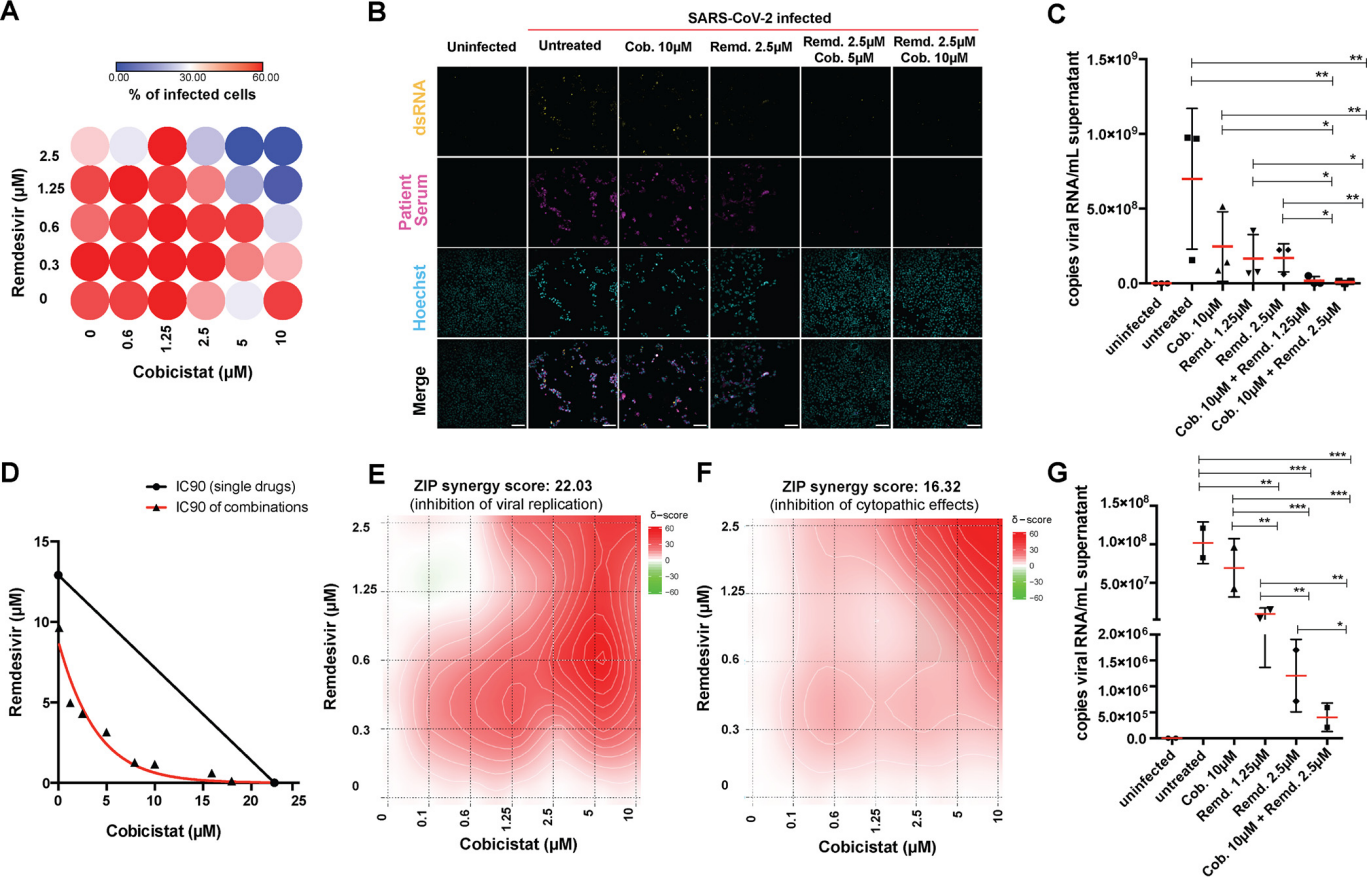
The combination of cobicistat and remdesivir synergistically inhibits SARS-CoV-2 activity. (A to F) Synergistic activity of cobicistat and remdesivir in inhibiting replication and cytopathic effects of SARS-CoV-2 in Vero E6 cells. Cells were infected at 0.5 MOI and left untreated or treated with the drugs at the indicated concentrations 2 h postinfection. Forty-eight hours posttreatment, cells were fixed for immunofluorescence (IF) staining (A and B), supernatants were collected for qPCR (C to E), or cellular viability was analyzed (F). For IF detection, cells were stained with sera of SARS-CoV-2 patients and with the J2 antibody, which binds to double-stranded RNA (36). The percentage of infected cells was determined by automatic acquisition of nine images per well (A), as described in Materials and Methods. Scale bar = 100 μm. Viral RNA in supernatants was detected by qPCR using an *in vitro*-transcribed standard curve for absolute quantification (C to E). Data, expressed as mean ± SD, were transformed as log_10_ to restore normality and analyzed by one-way ANOVA, followed by the Holm-Sidak posttest (C). Cellular viability was measured by MTT assay (F). Isobologram analysis of synergism (D) (82) was performed using the IC_90_ values for SARS-CoV-2 replication of cobicistat, remdesivir, or their combination, calculated by nonlinear regression. Synergism analyses of the inhibition of viral replication (E) or cytopathic effects (F) were performed with the SynergyFinder web tool (79) using the Zero Interaction Potency (ZIP) model based on inhibition values calculated as described in Materials and Methods. (G) Effect of the combination of cobicistat and remdesivir on SARS-CoV-2 RNA expression in supernatants of a primary human colon organoid. Treatment with cobicistat/remdesivir was performed 2 h postinfection, and supernatants were collected 48 h posttreatment. Viral RNA was quantified as described for panel C. For all panels, *n* equals 3 independent experiments, except for panel E (*n* = 2 independent experiments) and panel G (*n* = 2 replicates from one colon organoid donor). *, *P* < 0.05; **, *P* < 0.01; ***, *P* < 0.001.

While treatment with only remdesivir displayed antiviral activity at previously described levels (Fig. S8) (4), the combined use of cobicistat and remdesivir was able to significantly enhance the effect of each drug alone, in both cell lines (Fig. 5A to F; Fig. S9A to D). In particular, the drug combination was synergistic in almost completely abrogating viral infection/replication, as measured by IF (Fig. 5A and B; Fig. S9B) and qPCR (Fig. 5C to E; Fig. S9C). In line with this potent antiviral activity, the cobicistat-remdesivir combination also displayed a synergistic effect in inhibiting the cytopathic effects of SARS-CoV-2, thus restoring viability of infected cells to levels comparable to mock-infected controls (Fig. 5F; Fig. S9A and D). Finally, we tested the effect of the drug combination on a primary human colon organoid (Fig. 5G), which is susceptible to SARS-CoV-2 infection, as previously described (47). Also in this case, the addition of cobicistat enhanced the antiviral effect of remdesivir (Fig. 5G).

10.1128/mbio.03705-21.8 FIG S8 Antiviral effect of remdesivir on the Vero E6 and T84 cell lines. (A and B) Effect of serial dilutions of remdesivir on SARS-CoV-2 RNA amount in supernatants and on the viability of uninfected Vero E6 (A) and T84 (B) cells. Cells were left uninfected or infected with SARS-CoV-2 using an 0.5 MOI. Infected cells were left untreated or treated with remdesivir 2 hours postinfection. Forty-eight hours postinfection, supernatants were collected and viral RNA was assayed by qPCR. Cellular viability was measured by MTT assay in uninfected cells 48 h after treatment with different concentrations of remdesivir. Inhibition of viral replication and cell viability were normalized to the untreated control, and half-maximal inhibitory concentration (IC_50_) was calculated by nonlinear regression. Download FIG S8, PDF file, 0.4 MB. Copyright © 2022 Shytaj et al. 2022 Shytaj et al. https://creativecommons.org/licenses/by/4.0/ This content is distributed under the terms of the Creative Commons Attribution 4.0 International license.

10.1128/mbio.03705-21.9 FIG S9 Synergistic antiviral effect of cobicistat and remdesivir in the Vero E6 and T84 cell lines. (A to D) Effect of combined treatment with cobicistat and remdesivir on the viability of SARS-CoV-2-infected Vero E6 cells (A) and on viral replication (B and C) and inhibition of cytopathic effects (D) in T84 cells. Cells were infected at an 0.5 MOI and left untreated or treated with the drugs at the indicated concentrations 2 hours postinfection. Forty-eight hours posttreatment, cells were fixed for crystal violet (A) or immunofluorescence (IF) (B) staining, supernatants were collected for qPCR (C), or cellular viability was analyzed (D). For IF detection, cells were stained with sera of SARS-CoV-2 patients (B). Viral RNA in supernatants was detected by qPCR using an *in vitro*-transcribed standard curve for absolute quantification. Data, expressed as mean ± SD, were transformed as log_10_ to restore normality and analyzed by one-way ANOVA, followed by Holm-Sidak’s posttest (C). Scale bars = 100 μm. Cellular viability was measured by MTT assay, and synergism analysis of the inhibition of cytopathic effects was performed with the SynergyFinder web tool (79) using the Zero Interaction Potency (ZIP) model based on inhibition values calculated as described in Materials and Methods. *n* = 3 independent experiments (C and D). **, *P* < 0.01; ***, *P* < 0.001. Download FIG S9, PDF file, 0.2 MB. Copyright © 2022 Shytaj et al. 2022 Shytaj et al. https://creativecommons.org/licenses/by/4.0/ This content is distributed under the terms of the Creative Commons Attribution 4.0 International license.

Overall, our data prove that the combination of cobicistat and remdesivir can suppress viral replication in different cellular models of SARS-CoV-2 infection and suggest that cobicistat can exert a double activity as a direct antiviral and pharmacoenhancer.

### The combination of cobicistat and remdesivir decreases viral replication and disease progression in infected Syrian hamsters.

**FIG 6:**
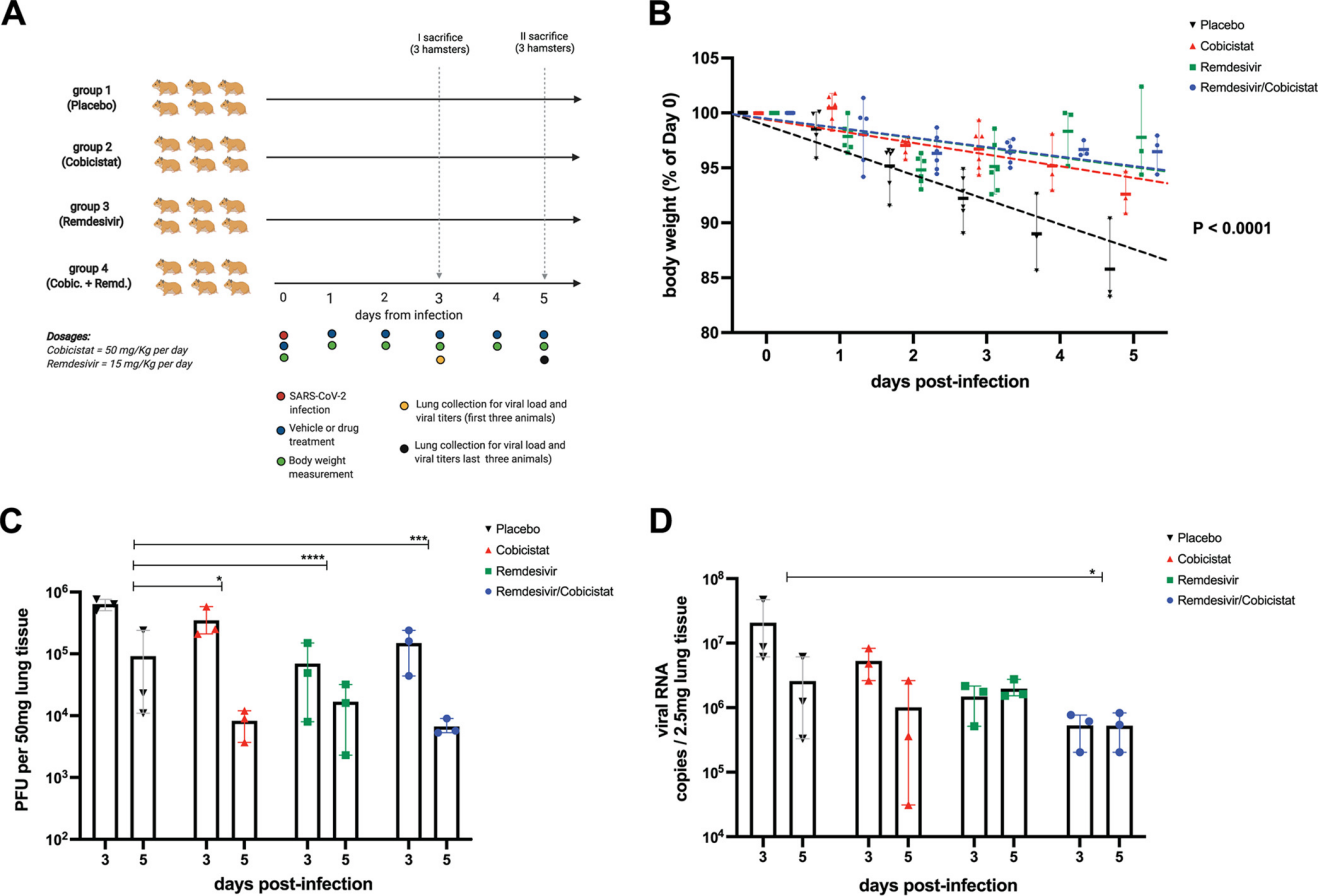
The combination of cobicistat and remdesivir inhibits SARS-CoV-2 replication and disease progression in Syrian hamsters. (A) Schematic representation (created with BioRender) of the *in vivo* dosing and sample collection of Syrian hamsters infected with SARS-CoV-2 and treated with placebo, cobicistat, remdesivir, or a combination of cobicistat and remdesivir. (B) Weight loss progression over time in the placebo and each treatment group. Data are expressed as the mean ± SD of the percentage over the baseline (day 0 postinfection [p.i.]) weight of each animal (*n* = 6 until day 3 p.i. and *n* = 3 at days 4 to 5 p.i.). Data were analyzed by linear regression for each experimental group, followed by the parametric F-test to assess differences among slopes. (C and D) Replication-competent viral titers as PFU on Vero E6 cells (C) and gRNA viral levels in the lung as measured at day 3 (*n* = 3) and 5 (*n* = 3) p.i. by plaque assay (C) and qPCR (D) quantification. Data were analyzed by two-way ANOVA followed by Tukey’s posttest, comparing the cumulative effects of treatments at both day 3 and day 5 p.i. Before the statistical analysis, an appropriate transformation was applied to make the results uniform (i.e., exponential transformation for PFU and standard log transposition for viral RNA copy numbers, due to the respective size-dependent restriction or amplification of the signal derived from the tests adopted). *, *P* < 0.05; ***, *P* < 0.001; ****, *P* < 0.0001.

We finally tested the *in vivo* effect of cobicistat, alone or in combination with remdesivir. To this purpose, we used Syrian hamsters, i.e., a well-validated animal model of SARS-CoV-2 infection mimicking several features of COVID-19 in humans (48, 49). The experimental setup (depicted in Fig. 6A) consisted of four groups of Syrian hamsters, which were infected with SARS-CoV-2 and then treated with either placebo, cobicistat (50 mg/kg daily), remdesivir (15 mg/kg daily), or a combination of the two drugs. For each treatment group, out of the total six animals, half of the hamsters were sacrificed at day 3 postinfection and the other half at day 5 postinfection in order to evaluate viral replication in lung at different stages of the disease. Overall, all treatment groups were characterized by a lower weight loss over time compared to the placebo group (slope difference *P* < 0.0001) (Fig. 6B). Moreover, in line with our *in vitro* data, cobicistat administration resulted in a significant decrease in the amount of infectious progeny in the lung (Fig. 6C). A similar and even more robust effect was observed upon treatment with remdesivir alone (Fig. 6C). Importantly, a combination of cobicistat and remdesivir was at least as effective as remdesivir alone in reducing the infectious viral progeny and also significantly decreased viral genomic RNA (gRNA) in the lung (Fig. 6C and D). In line with these results, several aspects of SARS-CoV-2-induced lung histopathology were attenuated in treated hamsters, particularly in the remdesivir and combination treatment groups (Table 5).

**TABLE 5:** Lung histopathological parameters in SARS-CoV-2-infected Syrian hamsters left untreated or treated with cobicistat, remdesivir, or their combination^*a*^

Treatment	Pneumonia						Vascular alterations						Alveolar alterations					
	Inflammation score		Consolidated lung area (%)		Lung area affected (%)		Endothelialitis		Perivascular edema		Perivascular lymphocytes		Type II hyperplasia		Alveolar edema		Epithelial necrosis	
	Day 3	Day 5	Day 3	Day 5	Day 3	Day 5	Day 3	Day 5	Day 3	Day 5	Day 3	Day 5	Day 3	Day 5	Day 3	Day 5	Day 3	Day 5
Placebo	2–3	3	15	50	40	80	2	3	2	2–3	(1)	2	1–2	2–3	(1)	3	2	2–3
	2–3	2–3	15	10	20	30	2–3	1–2	1	2–3	1	1	(1)	1–2	2–3	1–2	1	2
	2	3	10	30	20	90	1–2	3	(1)	2	(1)	2	1	4	1	2	1	2

Cobicistat	1	2–3	<5	35	5–10	50	1–2	3	1–2	2	0	1	0	1–2	0	2–3	(1)	1
	2–3	3	20	50	30	80	2–3	2–3	1–2	2–3	1–2	2	1–2	2–3	2	3	1	2
	(1)	3	0	30	<5	70	(1)	2–3	0	2	0	2	0	3	0	2	0	2

Remdesivir	(1)	2–3	0	25	5	40	0	2–3	(1)	(1)	0	2	0	1	0	1	(1)	2
	(1)	2–3	0	35	<5	70	0	2	(1)	1	0	1–2	0	1	0	1–2	0	2
	(1)	2–3	0	30	<5	40	0	2–3	0	2	(1)	2	0	1–2	0	2	0	2

Cobicistat + remdesivir	1	2	0	20	10	40	(1)	2	1	(1)	0	1	0	1–2	0	1–2	(1)	1
	(1)	2–3	0	30	5	50	0	2	(1)	1	0	1	0	2	0	1–2	0	1
	1	2–3	<5	15	5–10	40	0	2–3	(1)	1–2	0	2	0	1	0	1–2	(1)	2

a The experimental setup is depicted in Fig. 6A. Parameters were assessed at days 3 and 5 postinfection (three different animals at each time point) by investigators in a single-blind manner. 0 = absent, (1) = minimal, 1 = mild, 2 = moderate, 3 = severe, 4 = extreme.

Taken together, these results show that cobicistat, alone or in combination with remdesivir, has antiviral activity *in vivo* against SARS-CoV-2 infection.

## DISCUSSION

The data here presented demonstrate the antiviral activity of the FDA-approved drug cobicistat and support its possible role as a basis for combined antiviral therapies against SARS-CoV-2. The use of drug combinations targeting different steps of the viral life cycle is a well-established paradigm for treating RNA virus infections (6). Translating this concept to SARS-CoV-2 drug development has, however, proven challenging due to the paucity of effective drug candidates available. In particular, compounds showing promise in initial studies have failed to reproducibly decrease the mortality and morbidity of the infection (4, 5, 15). Similarly disappointing results were observed in the early stages of HIV-1 drug discovery and might be partially explained by the inability of candidate antivirals to reach *in vivo* concentrations sufficient to completely block viral replication.

The use of pharmacoenhancers such as cobicistat (24) could help overcome this limitation. While the present study exclusively focused on the combination of cobicistat and remdesivir, more than 30% of all drugs are metabolized by the main cellular targets of cobicistat (i.e., CYP3A4/5) (50). For example, the recently described SARS-CoV-2 inhibitor plitidepsin (51) is mainly metabolized by CYP3A4 *in vitro* (52). Therefore, it is conceivable that a synergistic effect similar to that described for remdesivir might be obtained by coupling cobicistat with other antiviral agents. In particular, the 3CL_pro_ inhibitor PF-07304814, currently undergoing clinical testing after showing promising *in vivo* antiviral activity (53), is administered with the CYP3A inhibitor ritonavir as a booster. As cobicistat is a more selective derivative of ritonavir (24), its administration could combine its pharmacoenhancer activity with a direct antiviral effect, which was here proven in multiple *in vitro* and *in vivo* models. In line with this, we observed the strongest synergistic effect with remdesivir, when cobicistat was used at concentrations around its IC_50_ levels, suggesting a combination of pharmacokinetic and pharmacodynamic effects. Of note, the concentration range in which cobicistat could inhibit SARS-CoV-2 replication was higher than that achievable through standard dosages (i.e., 150 mg/day) approved for treatment of HIV-1 infection (24). This potential drawback could be mitigated by the fact that cobicistat was previously shown to be well tolerated at much higher concentrations, both in mice and in humans. Indeed, plasma levels achievable with a single administration of high-dose (i.e., 400 mg/day) (33) cobicistat in humans are predicted to overlap concentrations displaying antiviral activity *in vitro*. Our dosing regimen *in vivo* (i.e., 50 mg/kg) was accordingly both well tolerated and effective in Syrian hamsters. These observations can also explain the limited effects or outright lack of success of early trials testing the HIV-1 protease inhibitor darunavir, boosted by a standard cobicistat dose (31, 32), as well as the general lack of effect of background antiretroviral treatments, including those containing cobicistat and its parent drug ritonavir, in preventing COVID-19 in people living with HIV (54). It is important to note that drug regimens for HIV-1 treatment are based on the assumption of long-term (or even lifelong) administration, while antiviral treatment for SARS-CoV-2 typically spans between 1 and 2 weeks. Thus, an increase of the cobicistat dosage in the context of an acute illness such as COVID-19 could be considered because its administration would be transient. On the other hand, ritonavir administration at a very high dose (i.e., 1,200 mg/day) can be associated, also in the short term, with increased liver enzyme and triglyceride levels, thus suggesting the need of safety data before increasing the dose of cobicistat in people with hepatic disease or chronic heart conditions (55). Moreover, combined administration of cobicistat and corticosteroids might be not advisable, as it could lead to dangerous pharmacokinetic interactions (56).

Another possible limitation of candidate antivirals for SARS-CoV-2 treatment is the inability to reach specific tissue reservoirs of the infection. Remdesivir is a case in point, due to its quick metabolization and poor intestinal absorption (57). Previous experience with HIV-1 protease inhibitors suggests that cobicistat might overcome this limitation (58), in line with the synergistic effect that we observed when treating primary colon organoid and T84 colon adenocarcinoma cells with the combination of cobicistat and remdesivir. Intriguingly, the tissue penetration and activity of cobicistat in the main sites of CYP3A expression (i.e., gut and liver) might be relevant also for the route of administration of remdesivir. Currently, remdesivir requires intravenous administration due to its extensive first-pass metabolism (59), but recent data on isolated liver microsomes indicate that cobicistat might improve its absorption, perhaps allowing oral formulation of the drug (22). Increasing the scalability of remdesivir might *per se* improve its therapeutic potential, as an early treatment of the infection might prevent hospitalization and development of severe COVID-19 (60), a stage where the efficacy of remdesivir is more likely, although not firmly established (15).

An important advantage of antiviral combinations is the possibility to target multiple steps of the viral life cycle. Despite the *in silico* docking results of our study and of other groups (27–29), our *in vitro* data demonstrate that cobicistat does not inhibit the enzymatic activity of 3CL_pro_. Our molecular dynamics analysis suggests that this discrepancy might be reconciled when conformational entropy (−TΔS) is included among the analysis parameters, which reduces the expected binding energy between cobicistat and 3CL_pro_. On the other hand, our *in vitro* results suggest an effect of cobicistat on S-glycoprotein maturation or function. Although our data prove that cobicistat can inhibit fusion mediated by the cleaved S-protein, experiments in infected cells leave open the possibility that the drug might also have an effect on the uncleaved S-protein. Further studies will therefore be required to precisely characterize the molecular mechanism of antiviral action of cobicistat.

One limitation of our work is that it does not specifically examine the metabolization of remdesivir in the presence or absence of cobicistat. In this regard, recent evidence on the metabolic conversion of remdesivir indicates that cobicistat might improve its stability (22). Moreover, our gene silencing experiments suggest that expression of the targets of cobicistat, CYP3A4 and P-gp, and perhaps CYP3A5, can blunt the antiviral effect of remdesivir. Interestingly, the silencing of P-gp was the most effective in increasing the antiviral activity of remdesivir. The lower affinity of cobicistat for P-gp compared to that for CYP3A (58) might partially explain why micromolar concentrations of cobicistat are needed to induce a strong synergistic effect with remdesivir. However, since our study did not assess protein levels of CYP3A4/5 and P-gp, it is possible that the synergistic effect of cobicistat might also be influenced by different expression of these proteins at baseline, or upon cobicistat treatment. Moreover, the antiviral benefit of the drug combination, compared to either cobicistat or remdesivir alone, was more evident *in vitro*, possibly due to the presence of different mechanisms of metabolization of remdesivir *in vivo* (e.g., hydrolases) (61).

It is currently unknown whether cobicistat can reach effective concentrations when administered at clinically acceptable doses in humans. While only clinical trials will be able to test this question, previous results on the tissue distribution and plasma concentrations of cobicistat in mice and humans, as well as our *in vivo* results in Syrian hamsters, suggest that effective concentrations of cobicistat can be achieved in the main sites of SARS-CoV-2 replication using well-tolerated doses (33). Interestingly, remdesivir was well tolerated when administered with antiretroviral therapy (ART) regimens containing standard doses of cobicistat (62). However, its administration with higher doses of cobicistat has not been tested in humans so far and will require special caution due to the potential nephrotoxic effects of remdesivir (63), which might be enhanced by boosting its activity, and to the potential hepatotoxicity induced by P-gp inhibition in the presence of remdesivir (19).

Overall, our study introduces cobicistat as a candidate for inhibiting SARS-CoV-2 replication *in vitro* and *in vivo* and for designing combination therapies aimed at blocking or reversing the onset of COVID-19.

## MATERIALS AND METHODS

### Virtual screening and molecular docking.

Identification of potentially active SARS-CoV-2 inhibitors with desirable absorption, distribution, metabolism, excretion and toxicity (ADME-Tox) properties was performed by structure-based virtual screening (SBVS) of DrugBank V. 5.1.5 (64) compounds targeting the three-dimensional (3D) structure of SARS-CoV-2 3CL_pro_. The analysis was focused on the substrate-binding site, which is located between domains I and II of 3CL_pro_. The binding site was identified using the publicly available 3D crystal structure (Protein Data Bank [PDB] ID: 6W63). Structures of the previously described noncovalent protease inhibitor X77 (65), natively cocrystallized with 3CL_pro_, were used as a reference for the identification of binding-site coordinates and dimensions for the virtual screening workflow, as well as for the docking validation of positions generated from the screening.

Protein structure analysis and preparation for docking were performed using the Schrödinger protein preparation wizard (Schrödinger Inc.). Missing hydrogen atoms were added, bond orders were corrected, and unknown atom types were assigned. Protein side chain amides were fixed using program default parameters, and missing protein side chains were filled in using the prime tool. All non-amino acid residues, including water molecules, were removed. Further, unrelated ligand molecules were removed and active ligand structures were extracted and isolated in separate files. Finally, the minimization of protein strain energy was achieved through restrained minimization options with default parameters. The centroids of extracted ligands were then used to identify the binding site with coordinates and dimensions extended for 20 Å stored as a Glide grid file. Drug screening was performed using the Glide software (66). High-throughput virtual screening (HTVS) was performed with the fastest search configurations. After postdocking minimization, the top-scoring 10th percentile of the output docked structures were subjected to the standard precision docking stage (SP). Then, active ligand structures were extracted and isolated in separate files. Finally, the top 10% scoring compounds were selected and retained only if their good scoring states were confirmed by Extra precision docking.

Remdesivir docking to CYP3A4, CYP3A5, and P-gp structures was performed to assess its capacity as a substrate/inhibitor for these proteins. CYP3A4, CYP3A5, and P-gp structures were collected from the Protein Data Bank (PDB), IDs 5VC0, 5VEU, and 6QEE, respectively, and were subjected to the same preparation steps described above. Native inhibitors were used for identification of binding sites; the centroid of the known inhibitor zosuquidar was used to identify the drug binding pocket of the P-gp protein structure. Further, cocrystallized ritonavir was used for identification of the drug binding pocket in both CYP3A4 and -5. Receptor grids were generated for protein structures, for both CYP3A4 and CYP3A5. The heme iron of the protoporphyrin ring was added as a metal coordination constraint, allowing metal-ligand interaction in the subsequent docking steps. Docking was performed using flexible ligand conformer sampling allowing ring sampling with a 2.5-kcal/mol window. Retained poses for the initial docking phase were set to 5,000 poses, and only 800 best poses per ligand were selected for energy minimization. Finally, postdocking minimization was carried out for 10 poses per ligand with a 0.5-kcal/mol threshold for rejecting minimized poses.

### Molecular dynamics.

Candidate ligand-receptor complexes derived from docking simulations were chosen for further computational analysis through molecular dynamics. The complexes examined included SARS-CoV-2 3CL_pro_ protein bound to cobicistat, darunavir, X77, nelfinavir, ritonavir, tipranavir, GC376, lopinavir, and MG-132. The complex with the native cocrystallized binding ligand, X77, obtained from the PDB structure with the code 6W63 was used for comparison in addition to the docked X77. The ligand-receptor complexes were protonated and processed via Molecular Operating Environment (MOE 2012; Chemical Computing Group). The AMBER 18 molecular dynamics package was used for the molecular dynamics simulations. The force field AMBER ff14SB (67) was used for the protein while the force field GAFF2 (68) was used for the ligands. Each complex was solvated in a cubic box extending 15 Å in each direction. The system was neutralized by the addition of Na^+^ ions followed by the addition of extra Na^+^ and Cl^−^ to bring the salt concentration to 150 mM. The system was energy minimized using a series of steepest-descent and conjugate gradient minimization steps followed by a series of constant-pressure equilibration runs under decreasing position restraints, from 5.0 to 0.1 kcal mol^−1^ Å^−2^. After that, an unrestrained production run of 30 ns was performed. All the dynamics were performed in the NPT ensemble at 310 K employing a Langevin thermostat and Berendsen barostat with a nonbonded cutoff value of 8.0 Å. The trajectories were saved every 10 ps and analyzed for root mean square deviation (RMSD) equilibration using CPPTRAJ (69). After that, the binding energy between the ligand and the receptor was estimated using the molecular mechanics/generalized-born surface area (MM/GBSA) method as implemented in AmberTools (70). Three hundred snapshots per nanosecond were used for the binding energy estimation. The IGB model 8 was used at a salt concentration of 100 mM. Other parameters were left at their default values. The entropic contribution was estimated using a previously described method of calculation of interaction entropy (35, 71).

### Cell lines and primary human organoids.

The following cell lines were used for infection and/or relative quantification of gene expression: Calu-3 (ATCC HTB-55), Caco-2 (ATCC HTB-37), T84 (ATCC CCL-248), and Vero E6 (ATCC CRL-1586). Primary organoids derived from human colon and ileum were seeded in 2D as described in reference 47. Culture conditions and susceptibility to SARS-CoV-2 infection have been previously described (47, 72).

### Virus stock production and infection.

Viral stocks used for infections were produced by passaging the BavPat1/2020 SARS-CoV-2 strain in Vero E6 cells, and the infectious titer was estimated by plaque assay, as previously described (36). Infection experiments were conducted using 2.5 × 10^4^ or 2.5 × 10^5^ cells per well in 96- and 12-well plates, respectively. Cell lines were infected at an 0.05 or 0.5 MOI in medium with low fetal calf serum (FCS) content (2%). Colon organoids were infected in a 24-well plate using 6 × 10^4^ PFU per well. Two hours postinfection, cells were washed twice in phosphate-buffered saline (PBS) and resuspended in complete medium.

### *In vitro* drug treatments.

The following compounds were tested to determine their effects on 3CL_pro_ activity, cytotoxicity, or inhibition of SARS-CoV-2 replication: cobicistat (sc-500831; Santa Cruz Biotechnology), remdesivir (S8932; Selleckchem Chemicals), tipranavir (sc-220260; Santa Cruz Biotechnology), nelfinavir mesylate hydrate (PZ0013; Sigma-Aldrich), darunavir and lopinavir (both obtained through the AIDS Research and Reference Reagent Program, Division of AIDS, NIAID), MG-132 (M8699; Sigma‐Aldrich), and GC376 (BPS Bioscience).

### RNA isolation and cDNA retrotranscription.

RNA extraction for *in vitro* experiments was performed on cell lysates or supernatants using the NucleoSpin RNA minikit for RNA purification (Macherey-Nagel, Düren, Germany) according to the manufacturer’s instructions. The concentration of RNA extracted from cell lysates was measured using a P‐class P 300 NanoPhotometer (Implen GmbH, Munich, Germany).

Retrotranscription to cDNA was performed with 500 ng of intracellular RNA or 10 μL of RNA from supernatants, using the high-capacity cDNA reverse transcription kit (Applied Biosystems, Foster City, CA, USA) following the manufacturer’s instructions.

For *in vivo* samples, RNA was extracted from 25 mg of lung homogenates and oropharyngeal swabs using the innuPREP virus RNA kit (Analytik Jena, Jena, Germany) according to the manufacturer’s instructions.

### SARS-CoV-2 RNA standard.

For the preparation of a viral RNA standard to use in qPCR for quantification of viral copies in supernatants, SARS-CoV-2 N sequence was reverse transcribed from total RNA isolated from cells infected with the SARS-CoV-2 BavPat1 stain using Superscript 3 and specific primers (TTAGGCCTGAGTTGAGTCA). The resulting cDNA was amplified and cloned into the pJET1.2 plasmid. Plasmid DNA (10 μg) was linearized by AdeI restriction enzyme digestion, and DNA was purified using the NucleoSpin gel and PCR cleanup kit (Macherey-Nagel, Düren, Germany). For *in vitro* transcription, T7 RNA polymerase was used as previously described (73). *In vitro* transcripts were purified by phenol-chloroform extraction and resuspended in RNase-free water. RNA integrity was confirmed by agarose gel electrophoresis.

### Animal experiments.

All animal experimentation was conducted in accordance with national and international guidelines for the care and humane use of animals and approved by the relevant state authority (Landesamt für Gesundheit und Soziales, Berlin, Germany; permit number 0086/20). Preparation of SARS-CoV-2 virus stock and intranasal infection of Syrian hamsters (Mesocricetus auratus) were described previously (49). Briefly, a SARS-CoV-2 wild-type isolate (BetaCoV/Munich/BavPat2-ChVir984-ChVir1017/2020) was grown and titrated on Vero E6 cells. To ensure genetic integrity, passage 3 stocks were genome sequenced, and results showed conformity with the published sequence (GenBank MT270112.1) and confirmed the presence of the furin cleavage site essential for *in vivo* pathogenesis. Male and female hamsters at 6 to 10 weeks of age were inoculated under anesthesia with 1 × 10^5^ PFU SARS-CoV-2 in a total volume of 60 μl cell culture medium. Beginning on the day of infection, animals were randomly assigned to 4 groups (*n *= 6 per group) and treated with 15 mg/kg remdesivir, 15 mg/kg remdesivir and 50 mg/kg cobicistat, 50 mg/kg cobicistat, or placebo once a day. Remdesivir was applied intraperitoneally (i.p.) as an aqueous solution, and cobicistat was applied orally as suspension in 10% dimethyl sulfoxide (DMSO), 40% corn oil, and 50% strawberry syrup. On days 3 and 5 following infection, three randomly assigned animals per group were terminated to prepare samples for downstream analyses. Histopathology and semiquantitative scoring of lesions were performed as previously described for SARS-CoV-2-infected Syrian hamsters (49).

### qPCR analysis.

Gene and/or viral expression from *in vitro* samples was analyzed by SYBR green qPCR using, for each reaction, 10 μL of SsoFast EvaGreen supermix (Bio‐Rad Laboratories, Hercules, CA, USA), 500 nM forward and reverse primer (0.1 μL each from 100 μM stock), 8.8 μL water, and 1 μL cDNA. The primers used are listed in Table 2. The qPCR was performed on a CFX96/C1000 Touch qPCR system (Bio‐Rad Laboratories, Hercules, CA, USA) using the following PCR program: polymerase activation/DNA denaturation at 98°C for 3 min, followed by 45 cycles of denaturation at 98°C for 10 s and annealing/extension at 60°C for 40 s and a final extension step at the end of the program at 65°C for 30 s. Gene expression data were normalized using the threshold cycle {2[−ΔΔ *C*(*T*)]} method (74), using the Tata-binding protein (*TBP*) gene as a housekeeper control.

For *in vivo* experiments, RNA extracts from 25 mg lung homogenates of Syrian hamsters were assayed and viral gRNA copies were quantified in 10% of the obtained eluate volume with a one-step reverse transcription-qPCR (RT-qPCR) using a standard curve prepared from serial dilutions of a bacterial SARS-CoV-2 clone and the NEB Luna Universal Probe one-step RT-qPCR kit (New England Biolabs, Ipswich, MA, USA). The assay was performed with the previously published TaqMan primers and probe (SARS-CoV-2 E_Sarbeco) on a StepOnePlus RealTime PCR system (Thermo Fisher Scientific, Waltham, MA, USA).

### Effect of cobicistat on replication-competent virus.

To assess the *in vitro* effect of cobicistat on replication-competent SARS-CoV-2, Vero E6 cells were seeded at 125,000 cells per well in 24-well plates and infected at an 0.05 MOI. Two hours postinfection, cells were washed twice in PBS and resuspended in complete medium and cobicistat was added at a concentration of 2.5, 5, or 10 μM in DMSO. A 10 μM DMSO-only mixture was used as a control. Supernatants were collected at 24 h postinfection and used to overlay cells in the plaque assay, as previously described (75). Briefly, Vero E6 cells were seeded into 24-well plates at 2.5 × 10^5^ cells/well. On the next day, six times 10-fold serial dilutions of infectious supernatants were prepared in complete Dulbecco modified Eagle medium (DMEM), the medium was removed from the plates, and 200 μl of the dilutions was added to the cells in duplicates. Infection was performed at 37°C for 1 to 3 h, followed by removal of supernatant and overlaying with 1 mL of minimum essential medium (MEM) containing 0.8% carboxymethylcellulose (Sigma-Aldrich) and incubation for 72 h at 37°C. Water-rinsed cell monolayers were stained for 15 to 30 min with 2.3% crystal violet solution (Sigma-Aldrich) and extensively washed with tap water, and plaques were manually counted after drying.

For *in vivo* experiments, 50 mg of lung tissue was homogenized using a bead mill (Analytik Jena, Jena, Germany), 10-fold serially diluted in MEM, and plated on Vero E6 cells in 12-well plates. The dilutions were removed after 2 h of incubation at 37°C, and cells were overlaid with 1.25% microcrystalline cellulose (Avicel; FMC BioPolymer, Hamburg, Germany) in MEM supplemented with 10% fetal bovine serum (FBS) and penicillin-streptomycin. Three days later, cells were formalin fixed and stained with crystal violet, and plaques were counted.

### Reprocessing of microarray and RNA-Seq data.

Microarray gene expression data for CYP3A4/5 and P-gp in different anatomical tissues or cell lines were retrieved from the Homo sapiens Affymetrix Human Genome U133 Plus 2.0 Array data set. Data were filtered by applying the criteria “Healthy sample status” and “No experimental treatment.” From the initial list, tissues with sample size <25 were filtered out. The anatomy search tool was used to plot log_2_ expression ratios of the tested genes. Gene expression data in cell lines were retrieved from the aforementioned microarray data set and from the transcriptome sequencing (RNA-Seq) “mRNA Gene Level Homo sapiens (ref: Ensembl 75)” data set. The cell line condition filter was used to refine the analysis and include exclusively cell lines susceptible to SARS-CoV-2 infection (i.e., T84, Caco-2, Calu-3, and A-549).

### Cell viability.

Cell viability was evaluated by the MTT [3-(4,5-dimethylthiazol-2-yl)-2,5-diphenyl tetrazolium bromide] assay and by crystal violet staining as previously described (76, 77). Briefly, the MTT assay was conducted using the CellTiter 96 nonradioactive cell proliferation assay (MTT) (Promega; Madison, WI, USA). Cells were plated in a 96‐well plate at a concentration of 3 × 10^6^ cells/mL in 100 μL of medium. The MTT solution (15 μL) was added to each well, and after 2 to 4 h, the reaction was stopped by the addition of 100 μL of 10% SDS. Absorbance values were acquired using an Infinite 200 PRO (Tecan, Männedorf, Switzerland) multimode plate reader at a 570-nm wavelength.

For crystal violet staining, cells were fixed in 6% formaldehyde and incubated with 0.1% crystal violet for 15 min. Unbound staining was then washed with H_2_O, and cells were imaged using a Nikon Eclipse Ts2-FL microscope.

### 3CL_pro_ fluorescence resonance energy transfer (FRET) assay.

The activity of 3CL_pro_ was measured by FRET assay (BPS Bioscience, San Diego, CA, USA) according to the manufacturer’s instructions and as previously described (34). Briefly, serial dilutions of test compounds and known 3CL_pro_ inhibitors were incubated in a 384-well plate with the 3CL_pro_ enzyme and its appropriate buffer, containing 0.5 M dithiothreitol (DTT). Wells without drugs or without 3CL_pro_ were used as positive control of 3CL_pro_ activity and blank control, respectively. After a 30-min incubation, the 3CL_pro_ substrate was added to each well and the plate was stored for 4 h in the dark. The fluorescence signal was acquired on an Infinite 200 PRO (Tecan, Männedorf, Switzerland) using an excitation wavelength of 360 nm and a detection wavelength of 460 nm. Relative 3CL_pro_ was expressed as percentage of the positive control after subtraction of the blank.

### Immunofluorescence.

For immunofluorescence (IF) staining, cells were seeded on iBIDI glass-bottom 96-well plates and infected with the BavPat1/2020 strain of SARS-CoV-2 at an MOI of 0.5. Cells were rinsed in PBS and fixed with 6% formaldehyde, followed by permeabilization with 0.5% Triton X-100 (Sigma) in PBS for 15 min. Cells were then blocked in 2% milk (Roth) in PBS and incubated with primary antibodies in PBS (anti-double-stranded RNA [anti-dsRNA] mouse monoclonal J2 antibody [Scicons], 1:2,000, and convalescent SARS-CoV-2 patient serum, 1:250). Afterward, cells were washed twice in PBS-0.02% Tween and incubated with secondary antibodies in PBS (1:1,000 anti-mouse 568, goat anti-human IgG-Alexa Fluor 488 [Invitrogen, Thermo Fisher Scientific] for detection of human immunoglobulins in serum and goat anti-mouse IgG-Alexa Fluor 568 [Invitrogen, Thermo Fisher Scientific] for dsRNA detection). Nuclei were counterstained with Hoechst 33342 (Thermo Fisher Scientific; 0.002 μg/mL in PBS) for 5 min, washed twice with PBS, and stored at +4°C until imaging.

### Microscopy and image analysis.

Cells were imaged using a motorized Nikon Ti2 widefield microscope or with a Nikon/Andor (CSU W1) spinning disc using a Plan Apo lambda 20×/0.75 air objective and a back-illuminated electron microscopy charge-coupled device (EM-CCD) camera (Andor iXon DU-888). The JOBS module was used for automatic acquisition of 9 images per well. Images were acquired in 3 channels using the following excitation (Ex)/emission (Em) settings: Ex 377/50, Em 447/60 (Hoechst); Ex 482/35, Em 536/40 (Alexa Fluor 488); Ex 562/40, Em 624/40 (Alexa Fluor 568). When the spinning disc was used, the excitation was performed with 405-nm, 488-nm, and 561-nm lasers.

Quantification of infected cells (expressed as percentage of total cells imaged per well) was performed using a custom-made macro in ImageJ (78). After camera offset subtraction and local background subtraction using the rolling ball algorithm, nuclei were segmented using automated local thresholding based on the Niblack method. Region of interest (represented by the ring [5 pixels wide] around the nucleus) was determined for each individual cell. Median signal intensity was measured in the region of interest in Alexa 488 (convalescent SARS-CoV-2 serum) and Alexa 568 (dsRNA) channels. Threshold for calling infected cells was manually determined for each individual experiment using the data from mock-infected cells. The same image analysis procedure and threshold were used for all wells within one experiment.

### Syncytium formation assay.

For the syncytium formation assay, Vero E6 cells (0.2 × 10^6^ cells/well) were seeded on coverslips in a 12-well plate 24 h before transfection. Cells were transfected using TransIT-2020 or TransIT-LT1 (Mirus) with 0.75 μg of pCDNA3.1(+)-SARS-CoV-2-S and 100 μl Opti-MEM per well. At 2 h posttransfection, cells were treated with cobicistat (final concentration of 1 μM, 5 μM, and 10 μM), sera of convalescent SARS-CoV-2 patients (1:500 or 1:100), or DMSO. At 24 h posttransfection, cells were washed twice with PBS and fixed in 4% paraformaldehyde (PFA) for 20 min at room temperature. After another washing step, cells were permeabilized in 0.5% Triton for 5 min at room temperature, washed, and blocked in 3% lipid-free bovine serum albumin (BSA) in PBS-0.1% Tween 20 for 1 h at room temperature. After washing, cells were stained with the primary rabbit polyclonal anti-SARS-CoV-2 spike glycoprotein antibody (1:1,000; Abcam) for 1 h at room temperature or overnight at 4°C. After washing, cells were incubated with the secondary Alexa Fluor 488 goat anti-rabbit IgG antibody (1:500; Life Technologies) for 1 h at room temperature. Cells were then washed again and incubated with 4′,6-diamidino-2-phenylindole (DAPI; 1:1,000; Sigma-Aldrich) for 1 min followed by washing with PBS and deionized H_2_O. Images were acquired with a Nikon Eclipse Ts2-FL inverted microscope. S-protein fluorescence intensity was measured by a plate reader (Infinite m200pro; Tecan). For quantification, the anti-SARS-CoV-2 spike signal was normalized to the cell nucleus signal. After background was subtracted using the signal of untransfected cells, the spike expression on the cell surface was measured on nonpermeabilized cells whereas the total spike expression was measured on permeabilized cells. Syncytia with three or more nuclei surrounded by the antibody staining were considered for the quantification. The edges of the antibody staining were overdrawn with the polygon selection tool in Image J (78).

### Cell fusion assay.

The effect of cobicistat on the interaction between the spike protein and human ACE2 was evaluated using a previously described fusion assay (40). Briefly, TZM-bl cells expressing the spike protein were incubated with 4-fold serial dilutions of cobicistat for 1 h. Target cells expressing the human ACE2 (h-ACE2) and the HIV-1 Tat protein were then mixed 1:1, and firefly luciferase activity was measured after 24 h.

### Western blotting.

For Western blot experiments on infected cells, 0.5 × 10^6^ cells were lysed in a buffer (20 mM Tris–HCl, pH 7.4, 1 mM EDTA, 150 mM NaCl, 0.5% Nonidet P‐40, 0.1% SDS, and 0.5% sodium deoxycholate) supplemented with protease and phosphatase inhibitors (Sigma‐Aldrich, Saint Louis, MO, USA). Lysates were supplemented with loading buffer, boiled at 95°C for 10 min, and sonicated for 5 min using a Bioruptor Plus sonication device (Diagenode, Liège, Belgium). Protein lysates were then run on a precast NuPAGE Bis‐Tris 4 to 12% SDS-PAGE gel (Thermo Fisher Scientific, Waltham, MA, USA) and transferred onto a nitrocellulose membrane (GE Healthcare, Little Chalfont, UK) using a Trans-Blot device for semidry transfer (Bio‐Rad Laboratories, Hercules, CA, USA). Membranes were blocked using the Li-Cor Intercept (PBS) blocking buffer (Li-Cor Biosciences, Lincoln, NE, USA) for 1 h at room temperature and incubated overnight at 4°C with the following primary antibodies diluted in blocking buffer plus 0.2% Tween 20: anti‐β‐actin (1:10,000), (Sigma‐Aldrich, Saint Louis, MO, USA), anti‐SARS-CoV-2 S-protein (rabbit; 1:1,000; ab252690; Abcam), anti‐SARS-CoV-2 N-protein (mouse; 1:1,000; AB_2827977; Sino Biological), and sera of SARS-CoV-2 convalescent individuals (1:200). Sera were collected as described in reference 36, following signing of informed consent by the donors, as well as ethical approval by Heidelberg University Hospital. After primary antibody incubation, membranes were washed three times with 0.1% PBS‐Tween and incubated for 1 h with the following fluorescence-conjugated secondary antibodies: IRDye 800CW goat anti-human IgG, IRDye 800CW anti-rabbit, IRDye 700CW anti-mouse (Li-Cor Biosciences, Lincoln, NE, USA). All secondary antibodies were diluted 1:15,000 in blocking buffer plus 0.2% Tween. After three washes with 0.1% PBS‐Tween and one wash in PBS, fluorescence signals were acquired using a Li-Cor Odyssey CLx instrument.

For Western blot experiments on transfected cells used in the syncytium formation assay, Vero E6 cells were washed with PBS and lysed in a buffer (2% SDS, 50 mM Tris-HCl, pH 7.4, supplemented with a protease inhibitor cocktail [Roche, Basel, Switzerland]). Lysates were boiled at 95°C for 10 min and run on a precast 4 to 15% mini-Protean TGX gel (Bio‐Rad Laboratories, Hercules, CA, USA) under reducing conditions and transferred onto a polyvinylidene difluoride (PVDF) membrane (Bio‐Rad Laboratories, Hercules, CA, USA) using the Trans-Blot-Turbo system for semidry transfer (Bio‐Rad Laboratories, Hercules, CA, USA). Membranes were blocked with 5% milk–Tris-buffered saline-Tween (TBS-T; 0.1% Tween 20) for 1 h at room temperature and incubated with the primary antibodies rabbit anti-SARS spike glycoprotein (1:1,000, ab252690) or mouse anti-glyceraldehyde-3-phosphate dehydrogenase (anti-GAPDH) (1:1,000, sc-47724) in 5% milk-TBS-T for 1 h at room temperature or overnight at 4°C. After primary antibody incubation, membranes were washed three times with TBS-T (0.1% Tween 20) and incubated with the secondary antibodies mouse anti-rabbit IgG-horseradish peroxidase (HRP) (1:1,000, sc-2357) or anti-mouse IgGk BP-HRP (1:1,000, sc-516102) in 5% milk-TBS-T for 1 h at room temperature. After incubation, membranes were washed three times and the Clarity Western enhanced chemiluminescence (ECL) substrate was added and incubated for 5 min at room temperature. The membranes were imaged with the Azure 400 imaging system.

### Gene expression silencing with siRNA.

For silencing of CYP3A4, CYP3A5, and P-gp expression, 1.25 × 10^4^ Vero E6 cells were initially seeded in 12-well plates 1 day before transfection. Transfection was performed with jetPRIME (Polyplus transfection) using a 40 nM siRNA end concentration of either ON-TARGETplus nontargeting control siRNA (Horizon Discovery) or ON-TARGETplus human p-gp1, Cyp3A4, or Cyp3A5 siRNA SMARTPool (Horizon Discovery) by mixing it with jetPRIME buffer and jetPRIME reagent according to the manufacturer’s instructions. The mix was incubated for 10 min at room temperature and added dropwise to the cells. Cells were then infected at an MOI of 0.05 48 h posttransfection. Two hours postinfection, cells were washed twice in PBS and resuspended in complete medium, after which remdesivir was added at a concentration of 0.5 μM in DMSO. At 24 h postinfection, cells were lysed, and RNA was collected and used to analyze gene expression by qPCR.

### Statistical analysis.

Data normality assumptions were tested by D’Agostino and Pearson normality test (for *n* > 3). Multiple-group comparisons were conducted by nonparametric Kruskal-Wallis test, followed by Dunn’s posttest, or by one-way or two-way analysis of variance (ANOVA) followed by Holm-Sidak or Dunnett posttests, respectively. Half-maximal inhibitory (IC_50_), effective (EC_50_), and cytotoxic (CC_50_) concentrations of the compounds were estimated by nonlinear regression after data normalization. For synergy and for IC_50_ calculation, the normalized relative inhibition values were calculated according to the formula % inhibition = 100 × [1 − (*X* − mock infected)/(infected untreated − mock infected)], where *X* is each given treatment condition. Data analysis was conducted using GraphPad Prism v6 (GraphPad Software, San Diego, CA, USA). Synergy scores were calculated with the SynergyFinder web tool (79) using the Zero Interaction Potency (ZIP) model (80).
