# Supplementary figures and images for: The FDA-Approved Drug Cobicistat Synergizes with Remdesivir To Inhibit SARS-CoV-2 Replication In Vitro and Decreases Viral Titers and Disease Progression in Syrian Hamsters
Source: mBio. 2022 Mar 1;13(2):e03705-21. doi: 10.1128/mbio.03705-21 (PMC8941859; doi:10.1128/mbio.03705-21)

**A**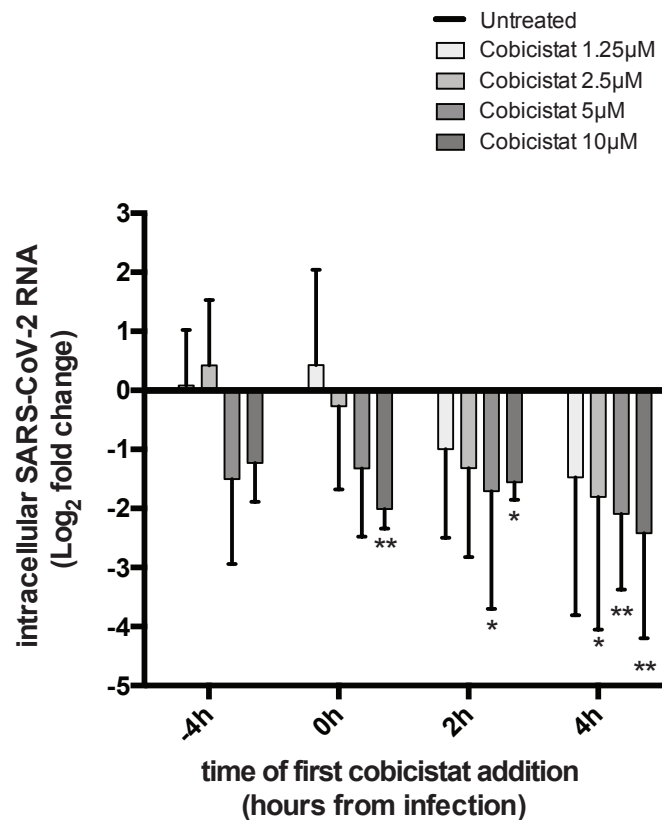**intracellular****B**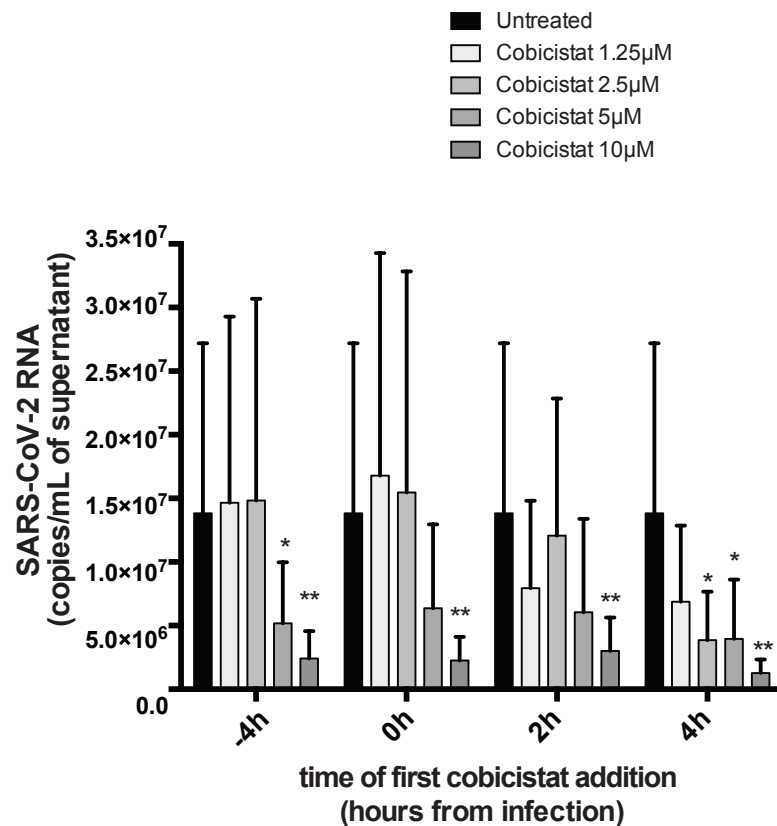**supernatant****C**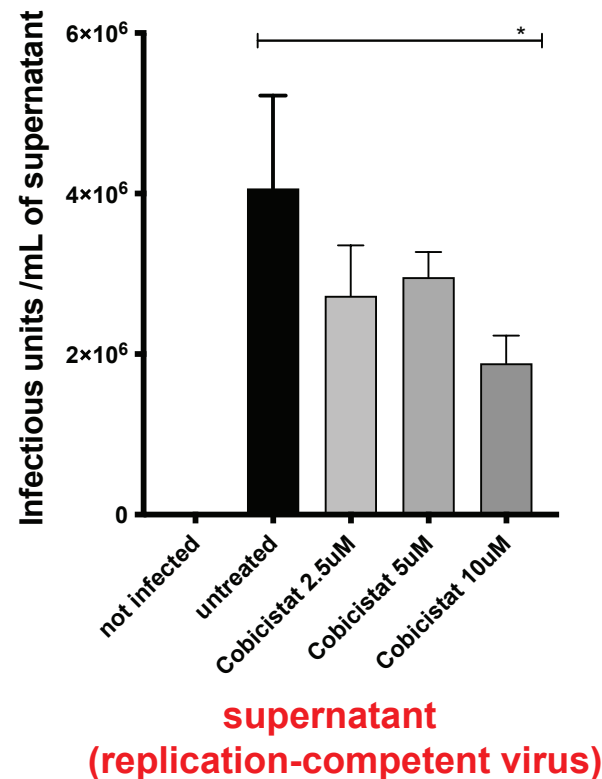**supernatant  
(replication-competent virus)**

Supplement: FIG S1 [file mbio.03705-21-sf001.pdf]

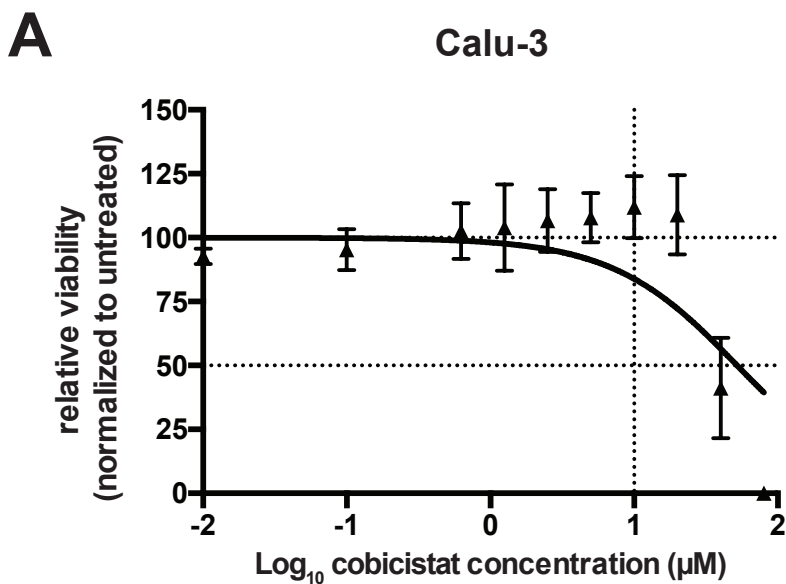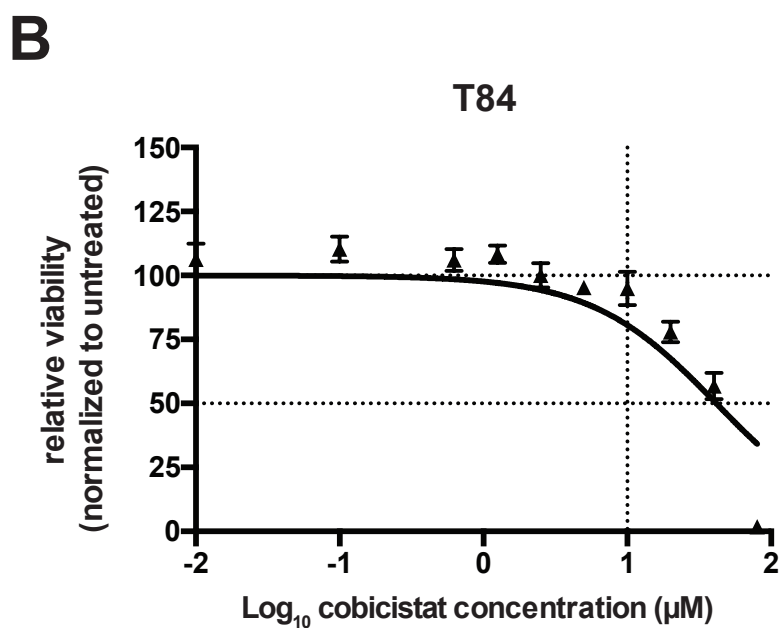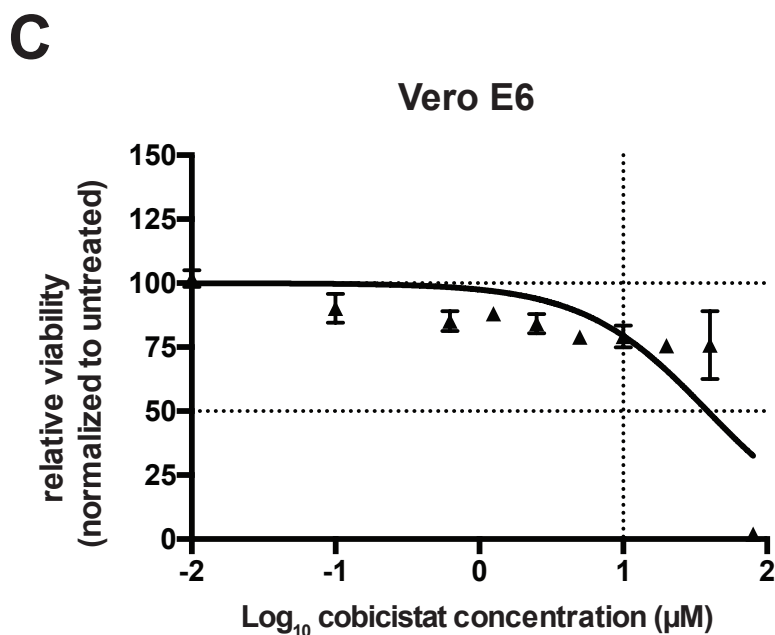

Supplement: FIG S2 [file mbio.03705-21-sf002.pdf]

**A**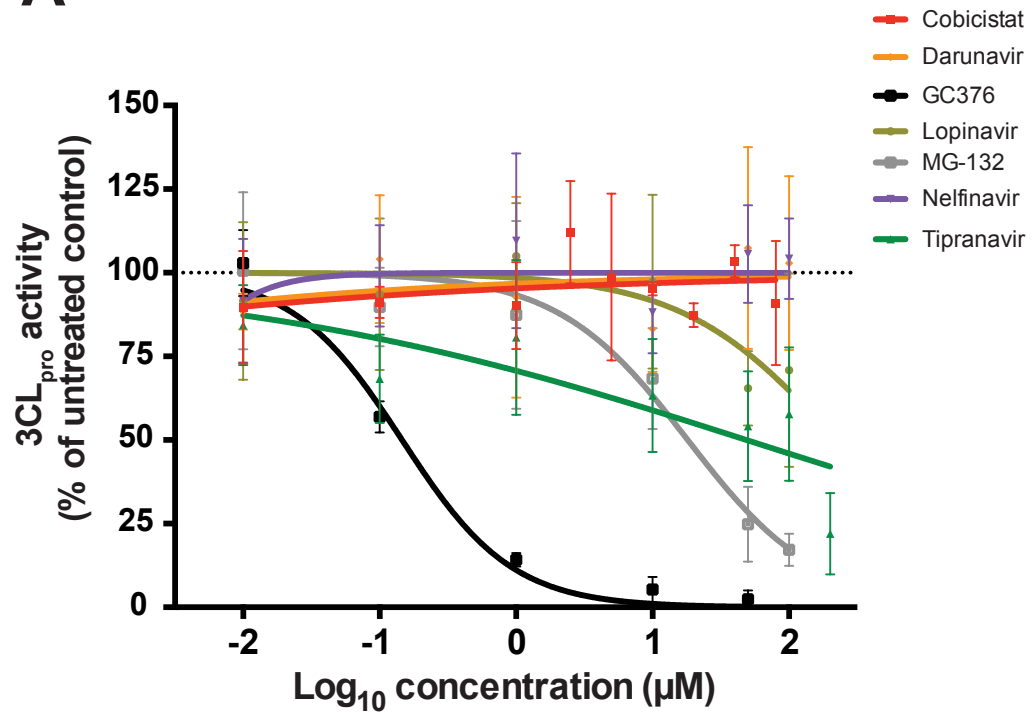**B**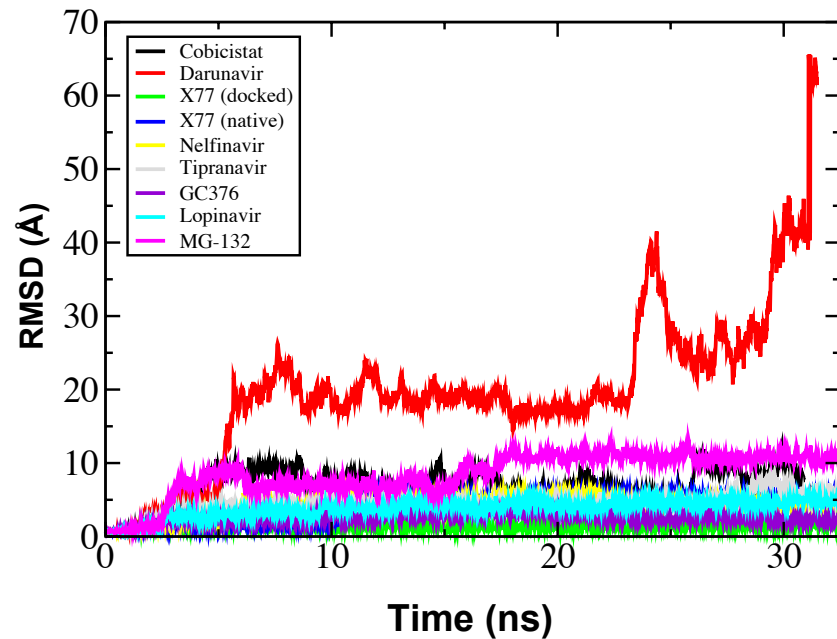

Supplement: FIG S3 [file mbio.03705-21-sf003.pdf]

**A**

SARS-CoV-2 infected

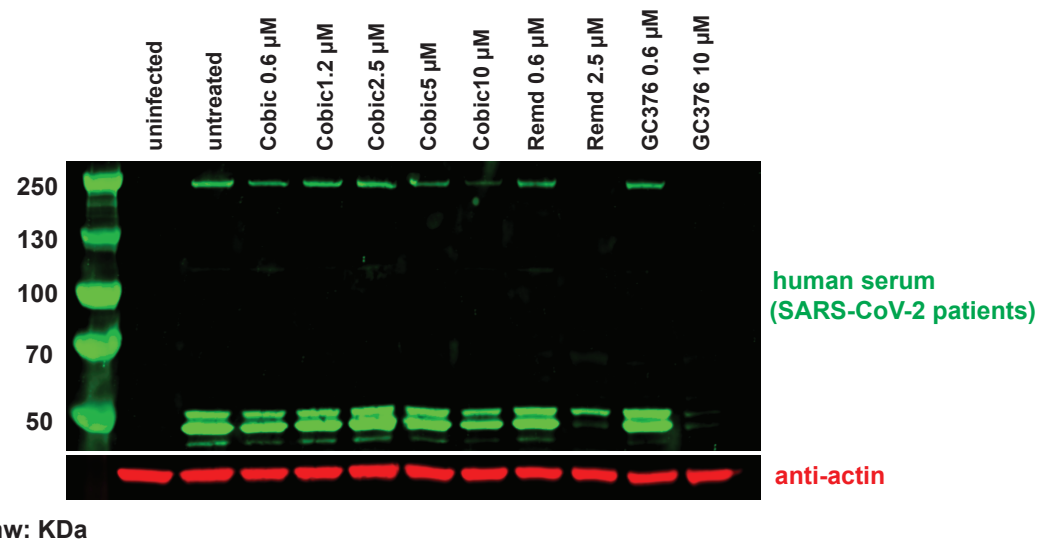**B**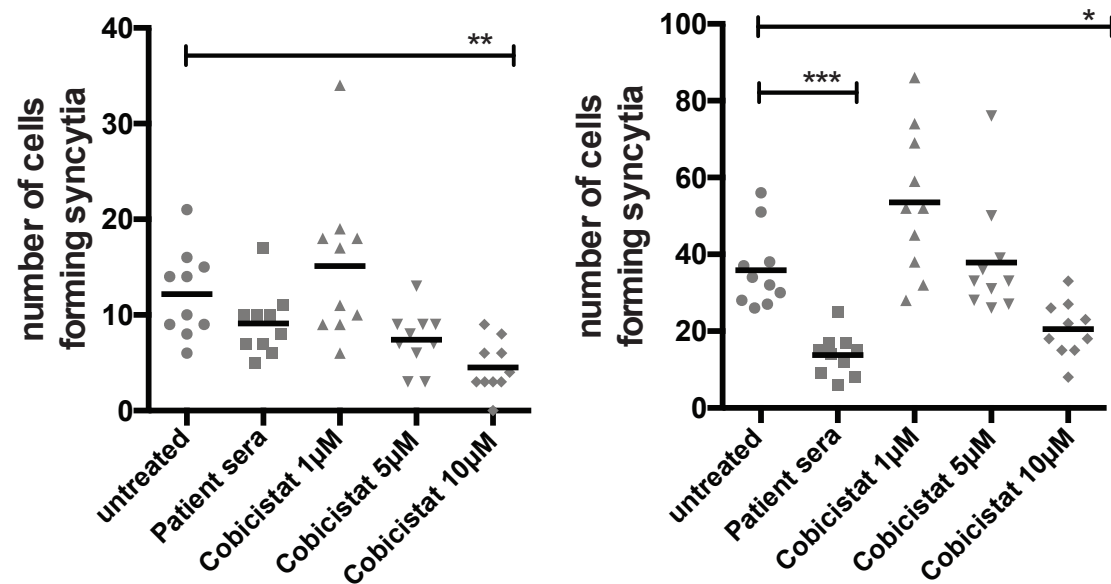**C**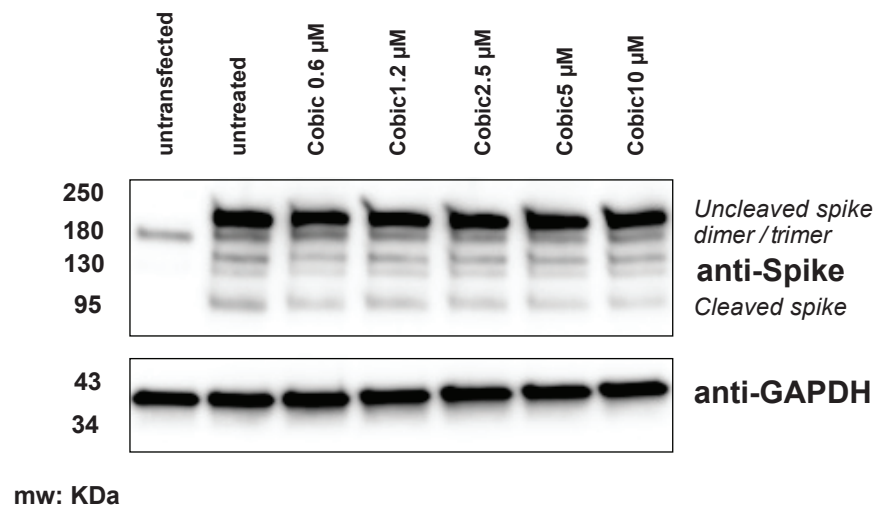**D**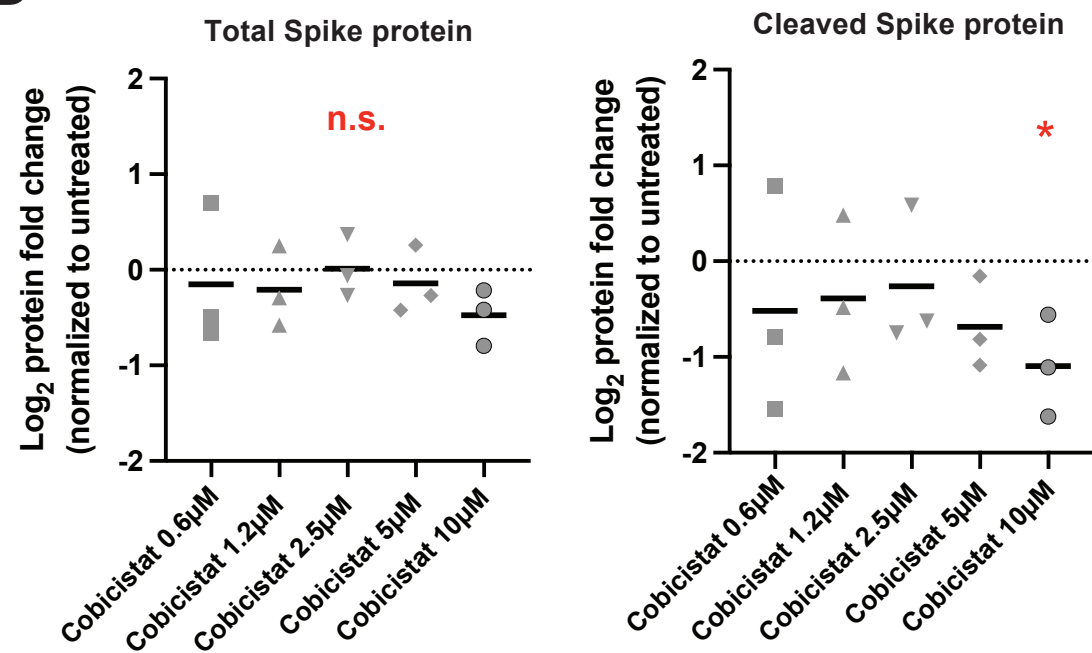**E**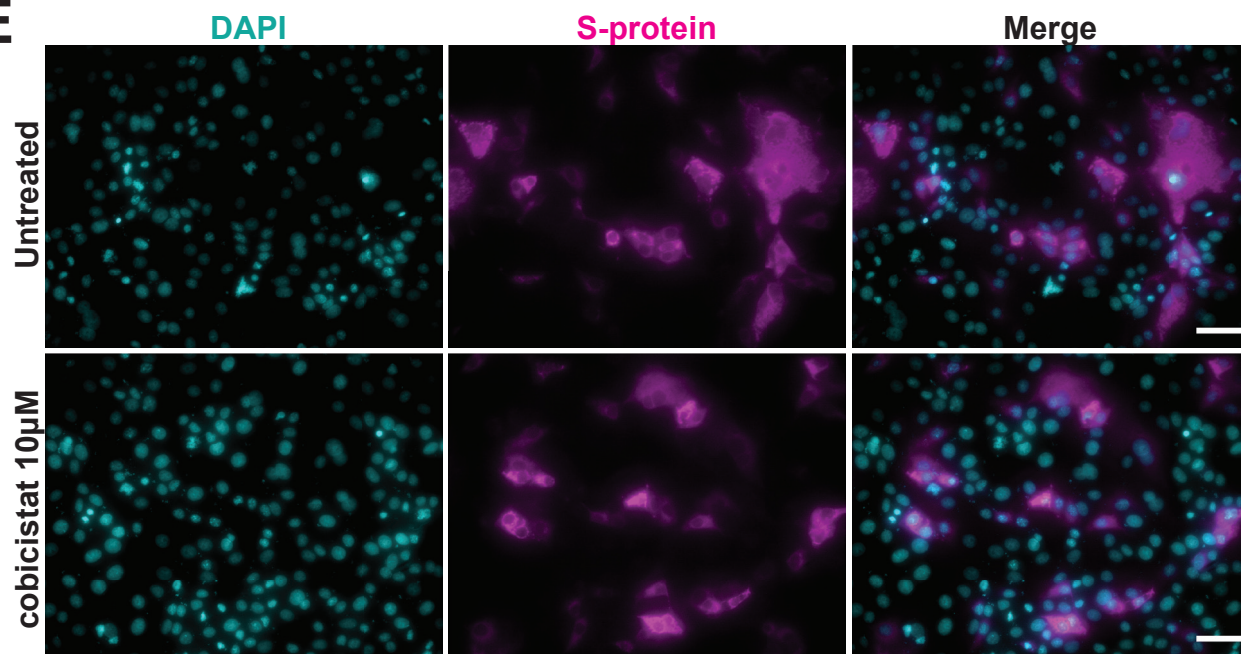**F**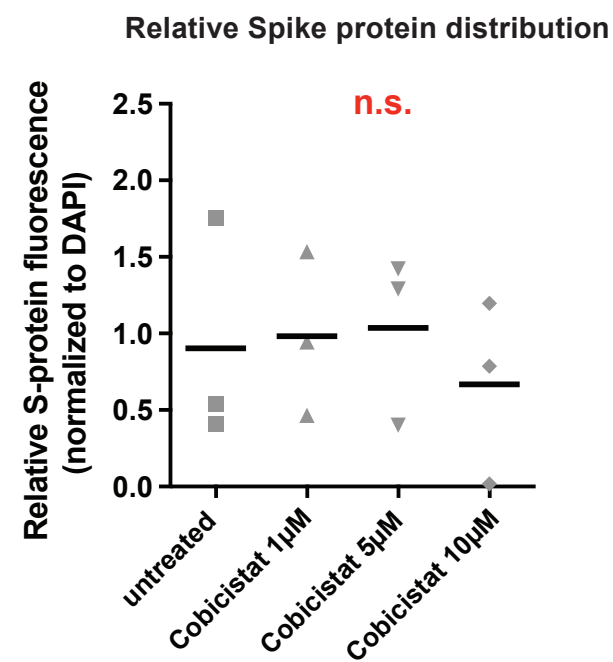

Supplement: FIG S4 [file mbio.03705-21-sf004.pdf]

*CYP3A4* gene expression

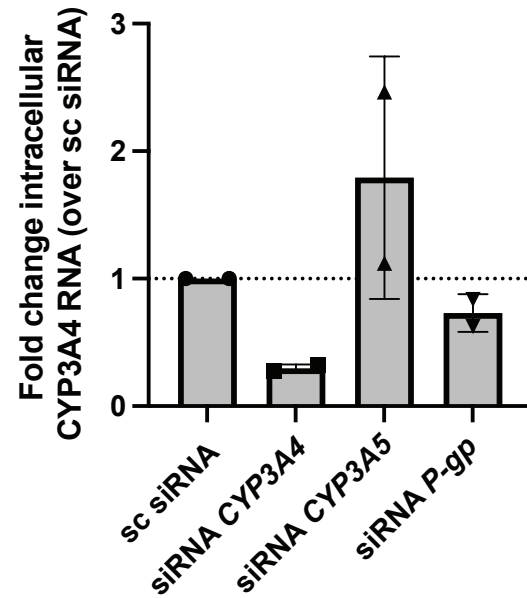

*CYP3A5* gene expression

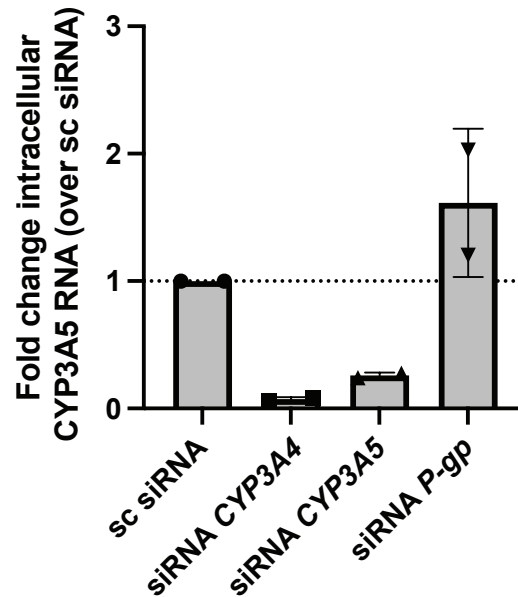

*P-gp* gene expression

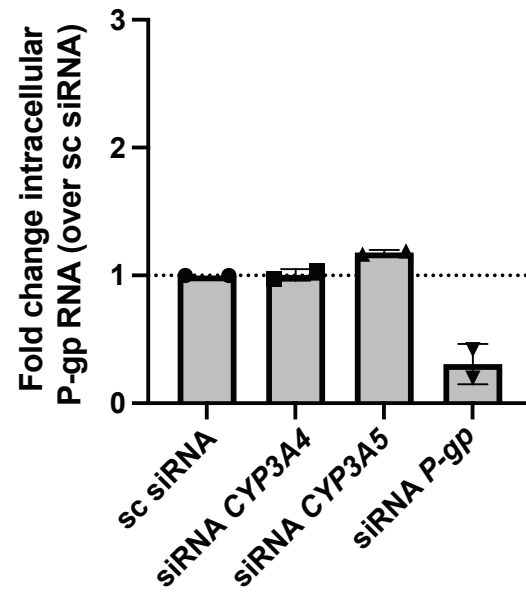

Supplement: FIG S6 [file mbio.03705-21-sf006.pdf]

**A**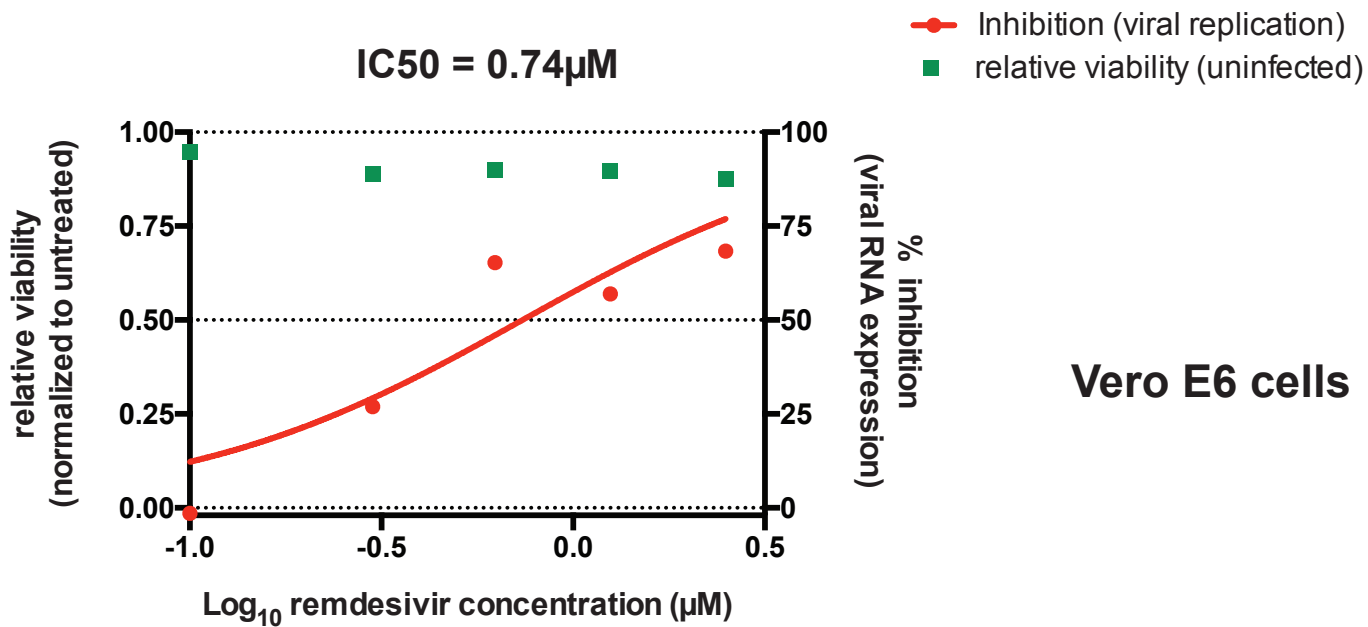**B**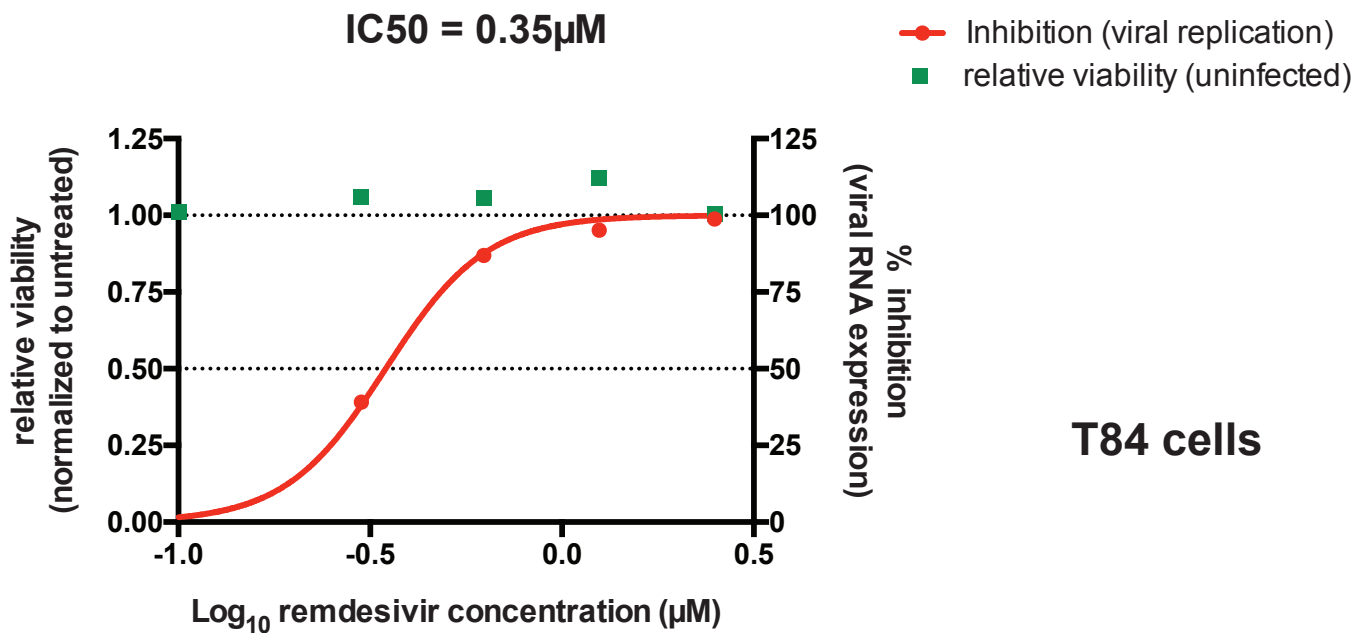

Supplement: FIG S8 [file mbio.03705-21-sf008.pdf]

**A**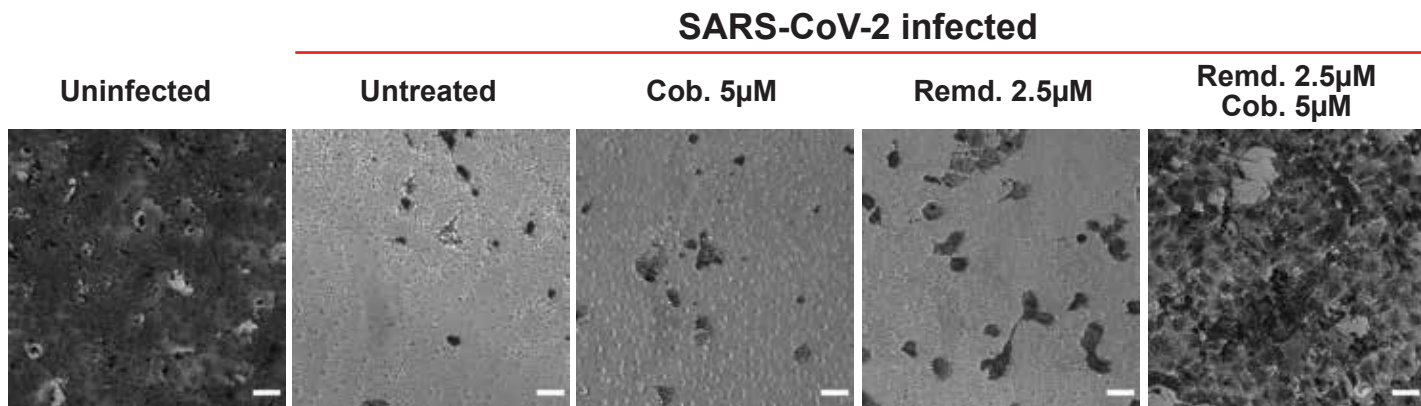**B**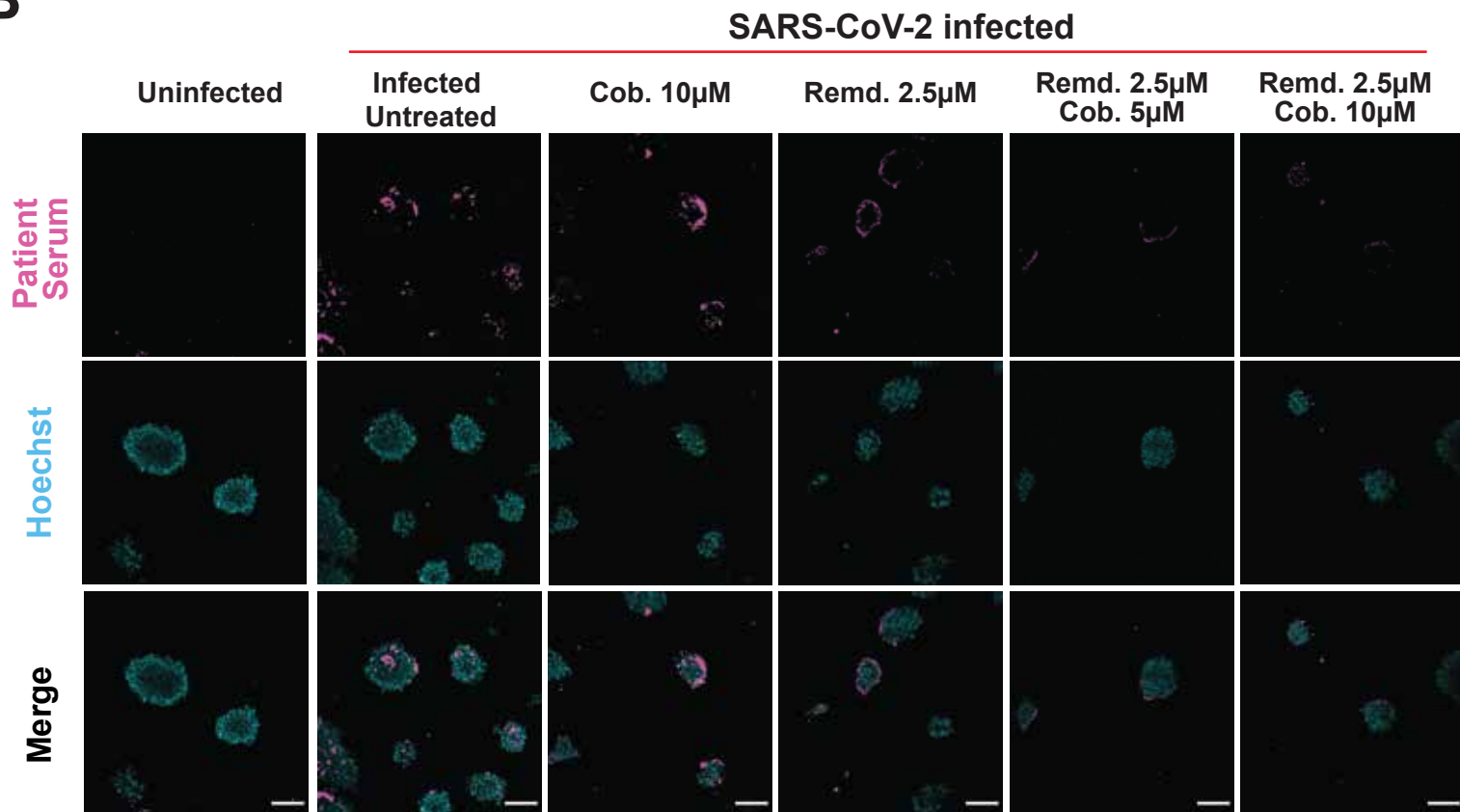**C**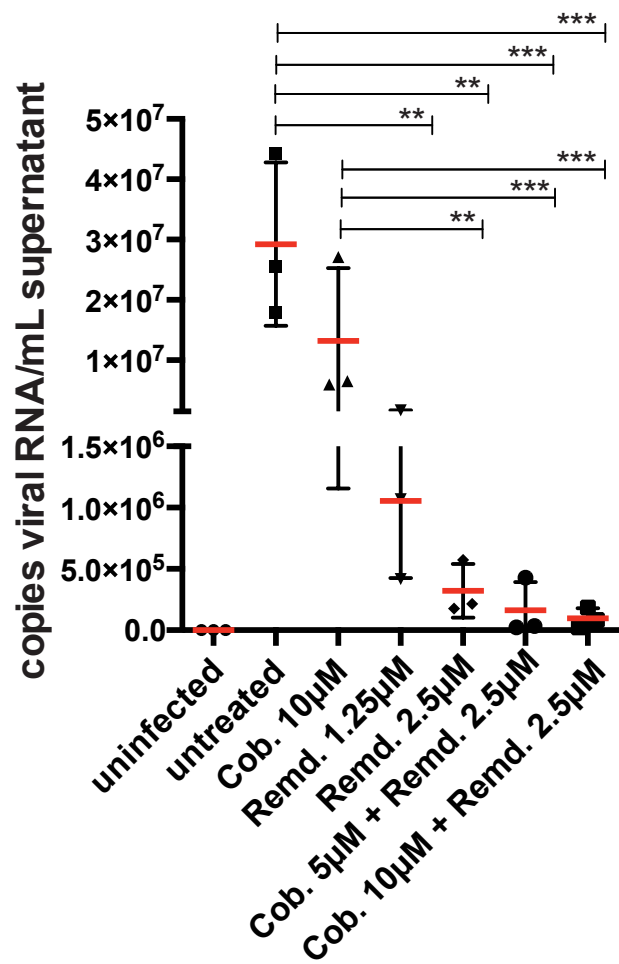**D**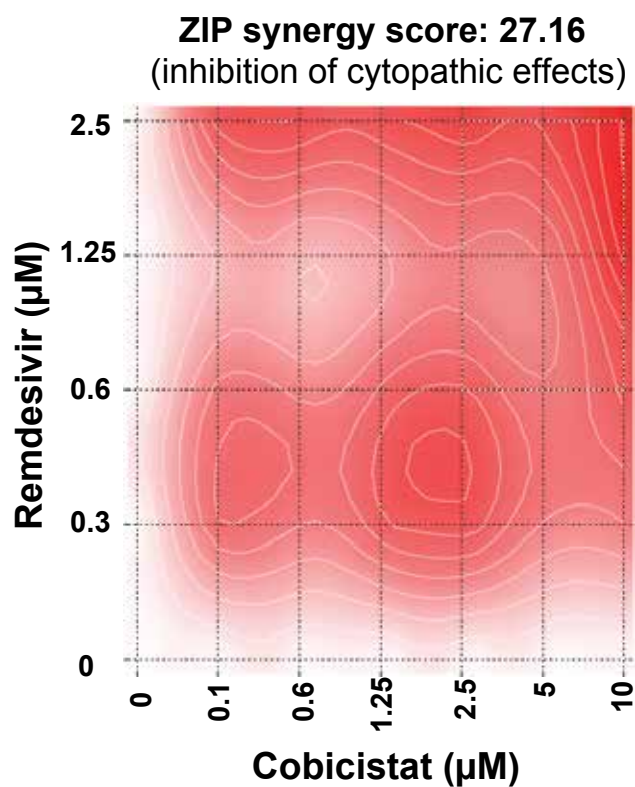

Supplement: FIG S9 [file mbio.03705-21-sf009.pdf]
