# Supplementary material for: The FDA-Approved Drug Cobicistat Synergizes with Remdesivir To Inhibit SARS-CoV-2 Replication In Vitro and Decreases Viral Titers and Disease Progression in Syrian Hamsters
Source: mBio. 2022 Mar 1;13(2):e03705-21. doi: 10.1128/mbio.03705-21 (PMC8941859; doi:10.1128/mbio.03705-21)
Supplement: FIG S5 [file mbio.03705-21-sf005.pdf]

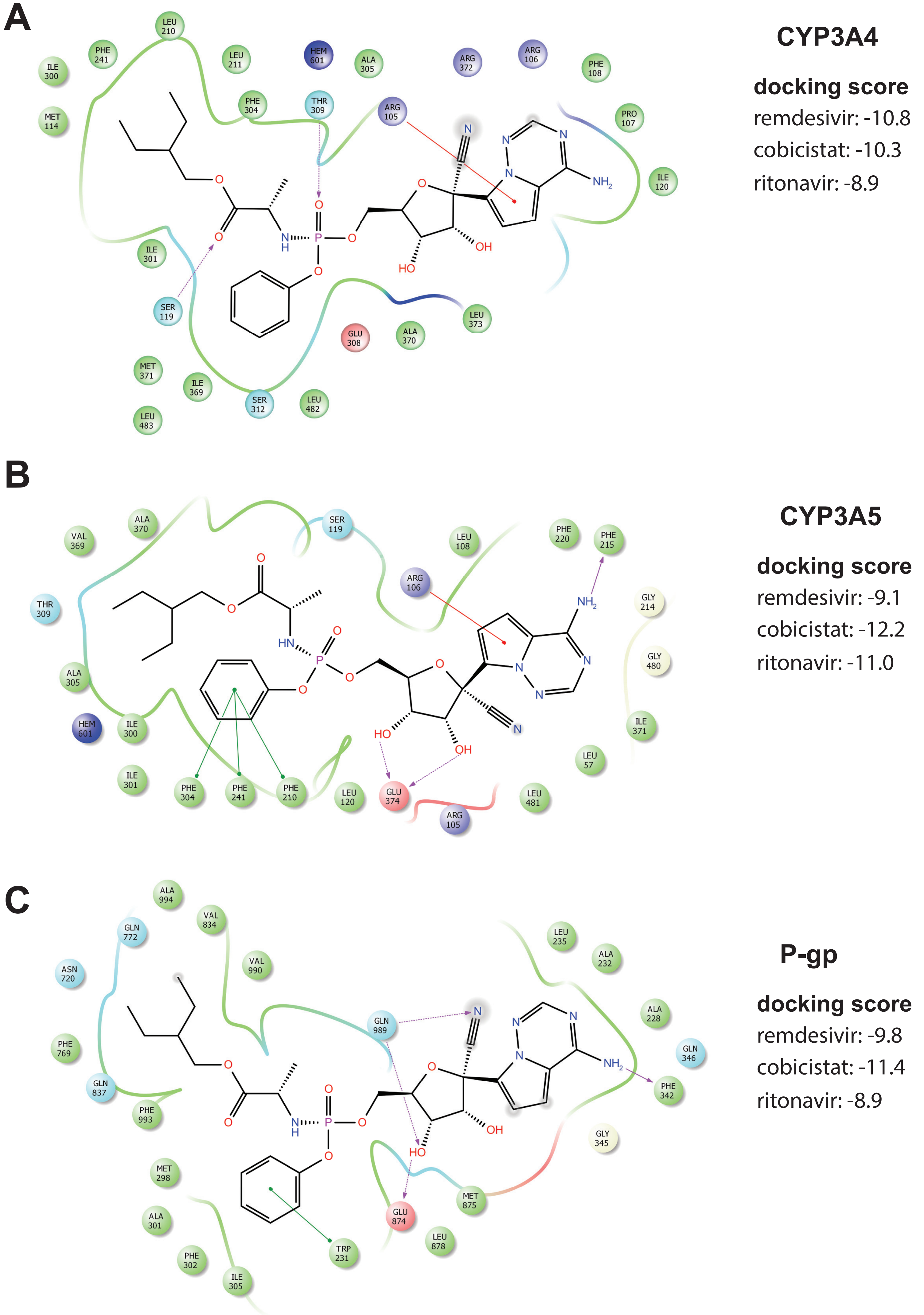
A
CYP3A4
docking score
remdesivir: -10.8
cobicistat: -10.3
ritonavir: -8.9
LEU 210
PHE 241
ILE 300
LEU 211
HEM 601
ALA 305
ARG 372
ARG 106
PHE 108
MET 114
PHE 304
THR 309
ARG 105
PRO 107
ILE 120
ILE 301
SER 119
GLU 308
ALA 370
LEU 373
MET 371
ILE 369
SER 312
LEU 482
LEU 483
B
CYP3A5
docking score
remdesivir: -9.1
cobicistat: -12.2
ritonavir: -11.0
VAL 369
ALA 370
SER 119
LEU 108
PHE 220
PHE 215
THR 309
ARG 106
GLY 214
GLY 480
ALA 305
HEM 601
ILE 300
ILE 371
LEU 57
ILE 301
PHE 304
PHE 241
PHE 210
LEU 120
GLU 374
ARG 105
LEU 481
C
P-gp
docking score
remdesivir: -9.8
cobicistat: -11.4
ritonavir: -8.9
ALA 994
VAL 834
GLN 772
ASN 720
VAL 990
LEU 235
ALA 232
GLN 989
ALA 228
GLN 346
PHE 769
GLN 837
PHE 993
PHE 342
GLY 345
MET 298
MET 875
GLU 874
LEU 878
ALA 301
TRP 231
PHE 302
ILE 305
