# Supplementary material for: The FDA-Approved Drug Cobicistat Synergizes with Remdesivir To Inhibit SARS-CoV-2 Replication In Vitro and Decreases Viral Titers and Disease Progression in Syrian Hamsters
Source: mBio. 2022 Mar 1;13(2):e03705-21. doi: 10.1128/mbio.03705-21 (PMC8941859; doi:10.1128/mbio.03705-21)
Supplement: FIG S7 [file mbio.03705-21-sf007.pdf]

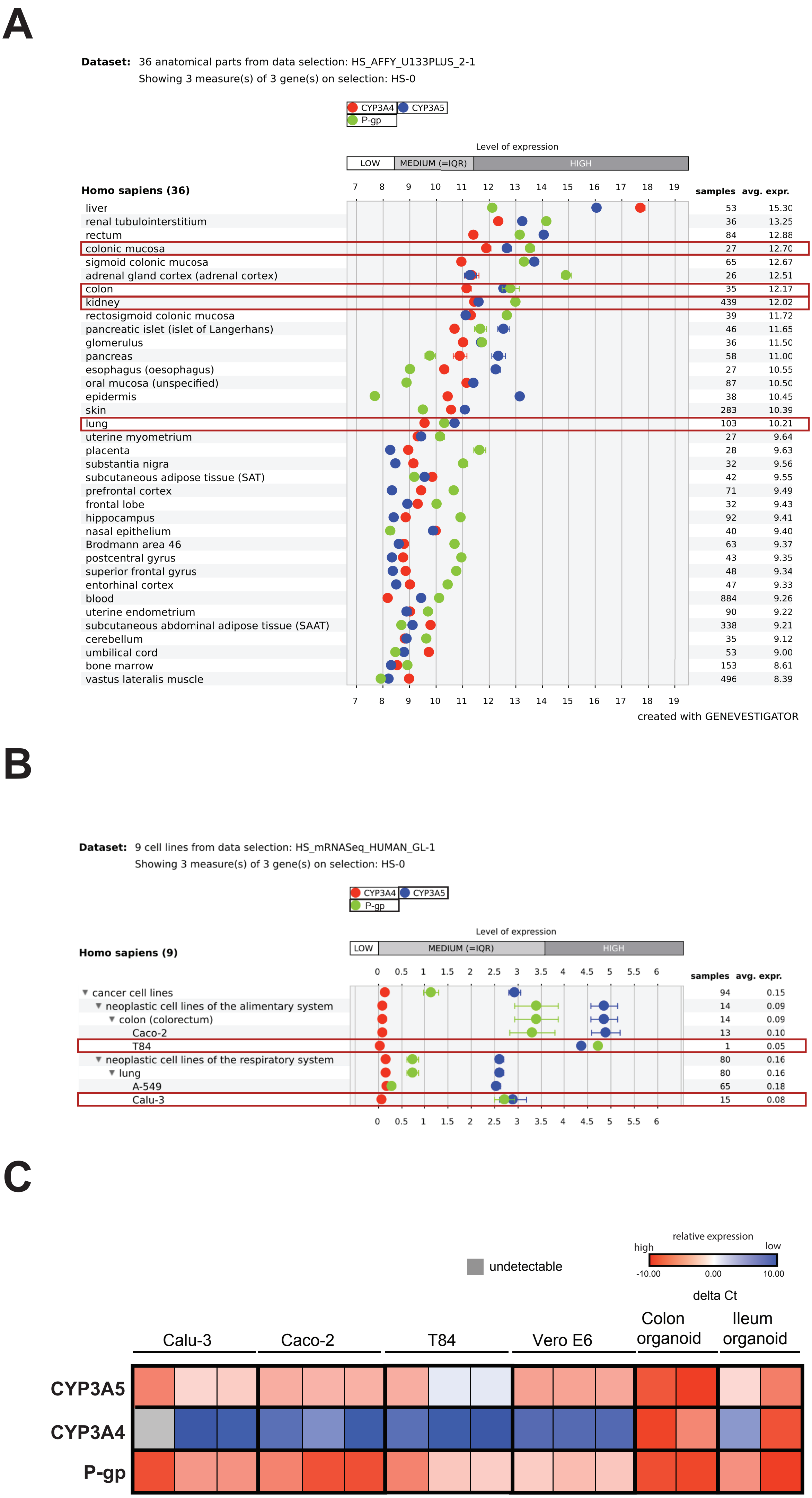
A
Dataset: 36 anatomical parts from data selection: HS_AFFY_U133PLUS_2-1
Showing 3 measure(s) of 3 gene(s) on selection: HS-0
CYP3A4
CYP3A5
P-gp
Level of expression
LOW
MEDIUM (=IQR)
HIGH
Homo sapiens (36)
samples
avg. expr.
liver 53 15.30
renal tubulointerstitium 36 13.25
rectum 84 12.88
colonic mucosa 27 12.70
sigmoid colonic mucosa 65 12.67
adrenal gland cortex (adrenal cortex) 26 12.51
colon 35 12.17
kidney 439 12.02
rectosigmoid colonic mucosa 39 11.72
pancreatic islet (islet of Langerhans) 46 11.65
glomerulus 36 11.50
pancreas 58 11.00
esophagus (oesophagus) 27 10.55
oral mucosa (unspecified) 87 10.50
epidermis 38 10.45
skin 283 10.39
lung 103 10.21
uterine myometrium 27 9.64
placenta 28 9.63
substantia nigra 32 9.56
subcutaneous adipose tissue (SAT) 42 9.55
prefrontal cortex 71 9.49
frontal lobe 32 9.43
hippocampus 92 9.41
nasal epithelium 40 9.40
Brodmann area 46 63 9.37
postcentral gyrus 43 9.35
superior frontal gyrus 48 9.34
entorhinal cortex 47 9.33
blood 884 9.26
uterine endometrium 90 9.22
subcutaneous abdominal adipose tissue (SAAT) 338 9.21
cerebellum 35 9.12
umbilical cord 53 9.00
bone marrow 153 8.61
vastus lateralis muscle 496 8.39
7 8 9 10 11 12 13 14 15 16 17 18 19
created with GENEVESTIGATOR
B
Dataset: 9 cell lines from data selection: HS_mRNASeq_HUMAN_GL-1
Showing 3 measure(s) of 3 gene(s) on selection: HS-0
CYP3A4
CYP3A5
P-gp
Level of expression
LOW
MEDIUM (=IQR)
HIGH
Homo sapiens (9)
samples
avg. expr.
cancer cell lines 94 0.15
neoplastic cell lines of the alimentary system 14 0.09
colon (colorectum) 14 0.09
Caco-2 13 0.10
T84 1 0.05
neoplastic cell lines of the respiratory system 80 0.16
lung 80 0.16
A-549 65 0.18
Calu-3 15 0.08
0 0.5 1 1.5 2 2.5 3 3.5 4 4.5 5 5.5 6
C
undetectable
relative expression
high
low
-10.00
0.00
10.00
delta Ct
Calu-3
Caco-2
T84
Vero E6
Colon organoid
Ileum organoid
CYP3A5
CYP3A4
P-gp
